# Multiproxy analysis of Upper Palaeolithic lustrous gravels supports their anthropogenic use

**DOI:** 10.1371/journal.pone.0291552

**Published:** 2023-11-01

**Authors:** Lila Geis, Francesco d’Errico, Fiona M. Jordan, Michel Brenet, Alain Queffelec

**Affiliations:** 1 CNRS, UMR5199, Pacea, Université de Bordeaux, Pessac, France; 2 Centre for Early Sapiens Behaviour (SapienCE), Department of Archaeology, History, Cultural Studies and Religion, University of Bergen, Bergen, Norway; 3 Department of Anthropology and Archaeology, University of Bristol, Bristol, England; 4 Inrap Nouvelle-Aquitaine Outre-mer, Pôle mixte de Recherches Archéologiques, Campagne, France; Sapienza University of Rome: Universita degli Studi di Roma La Sapienza, ITALY

## Abstract

Upper Palaeolithic sites in southwestern France attributed to the Upper Gravettian and the Solutrean yielded sub spherical gravels with a highly shiny appearance that have intrigued researchers since the 1930s. In this work, we analyze specimens from five sites, including the recently excavated Solutrean site of Landry, to establish whether their presence in archaeological layers and peculiar aspect are due to natural processes or human agency. We study the spatial distribution of gravels at Landry and submit archaeological gravels from the five sites, natural formations, Landry sediment sieving, and polishing experiments with a rotary tumbling machine to morphometric, colorimetric, microscopic, and textural analyses. Our results indicate the lustrous gravels found at the five sites result from deliberate selection and suggest their shiny appearance is the consequence of human agency, possibly resulting from prolonged contact with a soft material such as animal skin. Ethnographic accounts indicate that these gravels may have been used for magico-religious ritual purposes (charms, sorcery, divination etc.), in games, as elements of musical instruments, and as items serving other social and personal purposes. We argue that these objects reflect a cultural innovation emerged during the Gravettian and continued into the Solutrean.

## Introduction

**Fig 1:**
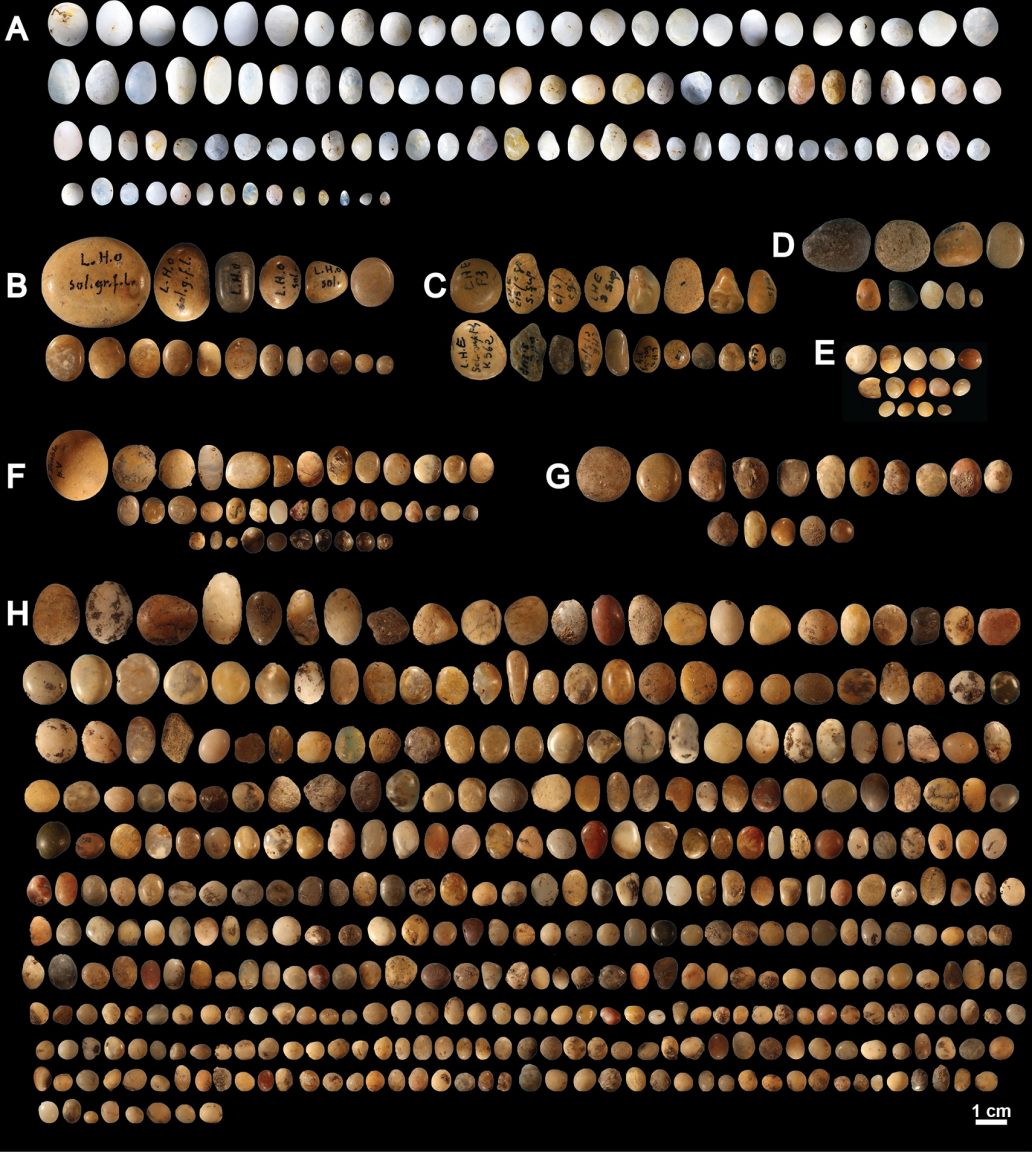
Lustrous gravels discovered in several Palaeolithic sites in southwestern France. (A) Late Solutrean gravels from Landry; (B): Late Solutrean gravels from Laugerie Haute West; (C) Late Solutrean gravels from Laugerie Haute East; (D) Solutrean gravels from Pech de la Boissière; (E) Solutrean gravels from the Abri Casserole; (F) Gravettian gravels from Fourneau du Diable; (G) Solutrean gravels from Fourneau du Diable and (H) Late Solutrean from Fourneau du Diable.

Upper Palaeolithic sites in southwestern France attributed to the Upper Gravettian and the Solutrean have yielded sub spherical gravels with a highly shiny appearance that have attracted the attention of Palaeolithic archaeologists since the 1930s [1–7]. Peyrony [6] thought that they were attached on a leather tag with a resin and arranged next to each other to be used as ornaments (Fig 1). Other researchers have limited themselves to reporting their discovery without attempting to determine whether their presence in archaeological layers and their shine were due to natural processes or human activity.

Answering these questions has been made difficult by the fact that these gravels were uncovered during excavations for which we lack relevant contextual data. We do not know, for example, whether gravels were systematically collected nor do we have precise information on their spatial distribution and eventual association with other archaeological remains. The discovery of about a hundred lustrous gravels on the recently excavated Solutrean site of Landry (Dordogne, France) (rescue excavation by Inrap in 2011, Brenet dir.), and the application of new analytical methods make it possible to approach the question of the origin and eventual anthropogenic modification of these objects in new ways.

In order to address this topic we combine in our study four approaches: 1) spatial analysis of lustrous gravels and their relationship with other archaeological remains unearthed at Landry, 2) morphometric and colorimetric analysis of gravels discovered at this site, four other Upper Palaeolithic sites from Dordogne, and gravels from geological deposits accumulated by known sedimentary processes, 3) surface texture analysis of lustrous and non-lustrous gravels from archaeological sites and natural accumulations as well as gravels experimentally polished to mimic natural and anthropogenic actions, 4) microscopic analysis of the natural, archaeological and experimental gravels, and 5) a survey of ethnographically-attested uses of gravels and small pebbles. The objective of the spatial analysis was to establish whether the gravels’ distribution was random or not, and connected with that of other archaeological remains. The morphometry and colorimetry of the lustrous and non-lustrous gravels aimed at verifying whether the former represents a selection of the gravels naturally available on the site. The textural analysis of archaeological, natural and experimentally polished gravel was intended to document and quantitatively compare their wear patterns to establish whether shine on archaeological objects could be found in nature, and which experimental polishes generated by a tumbler and a metallographic polisher better mimic archaeological wear. The microscopic analysis, conducted at different magnifications, was aimed at a more finely tuned characterization of natural, archaeological and experimental gravel’s surface conditions and intra-site comparisons.

The last decade has seen a substantial development of studies applying confocal microscopy and roughness parameters to archaeological artifacts with the objective of understanding the function of tools, the intensity of use, and taphonomic processes affecting the artefact after its production and use [8–17]. The present study is the first in which this approach is combined with others to establish whether the presence of objects in archaeological layers is the consequence of human action and to infer possible function just from wear. Implementation of this holistic approach leads us to conclude that the lustrous gravels found at the five sites result from deliberate selection and their shiny appearance is the probable consequence of prolonged contact with animal skin. The characteristic shine recorded on the archaeological gravels may have been produced deliberately or be the consequence of transport or use of the gravels. A survey of historical and ethnographic sources allows us to explore, for these three cases, what functions these gravels may have had and to suggest a general strategy for how to approach the study of archaeological objects presenting minimal modifications but potentially reflecting significant cultural innovations.

## Archaeological context

Lustrous gravels are reported from four sites of the South-West of France: Fourneau du Diable, Casserole, Pech de la Boissière and Laugerie Haute (Fig 1 and Table 1). The recent excavation at a fifth site, Landry, has for the first time yielded these objects in a stratigraphically secure and culturally well assigned context.

**Table 1:** Contextual data on Upper Palaeolithic sites with lustrous gravels.

Site	Type	Culture	Ages (BP)	Layer	NB	Sedimentary processes	Excavator(s)	Year(s)
**Landry**	Open air	Late Solutrean	21000	Upper level, sector 3	106	Solifluxion, acid attacks	Brenet	2012
**Fourneau du Diable**	Rock-shelter	Gravettian	29000–22000	Lower terrace	40	Screes before/after occupations, erosion, colluvium, sedimentary reorganization	Peyrony	1912–1929
		Solutrean	26000–23000	Lower terrace	16			1912–1929
		Late Solutrean	24000–23000	Upper terrace	409		Baumann	2015
**Pech de la Boissière**		Solutrean	26000–23000	No information	30	Screes, surface runoff	Peyrony	1929–1934
**Casserole**		Solutrean	23300–20500	No information	14	Block collapse, trampling, percolation, bioturbation, gelifraction	Kelley, Chadoune, Breuil, Dutrain	1939, 1991–1992
**Laugerie Haute**		Late Solutrean	23500–19600	East sector, layer 25	19	Block collapse, trampling, percolation, bioturbation, gelifraction	Lartet-Christy, Massenat-Girod, Capitan, Peyrony, Bordes, Smith	1863, 1892, 1895, 1921–1935, 1950
				West sector	18			

### Landry

**Fig 2:**
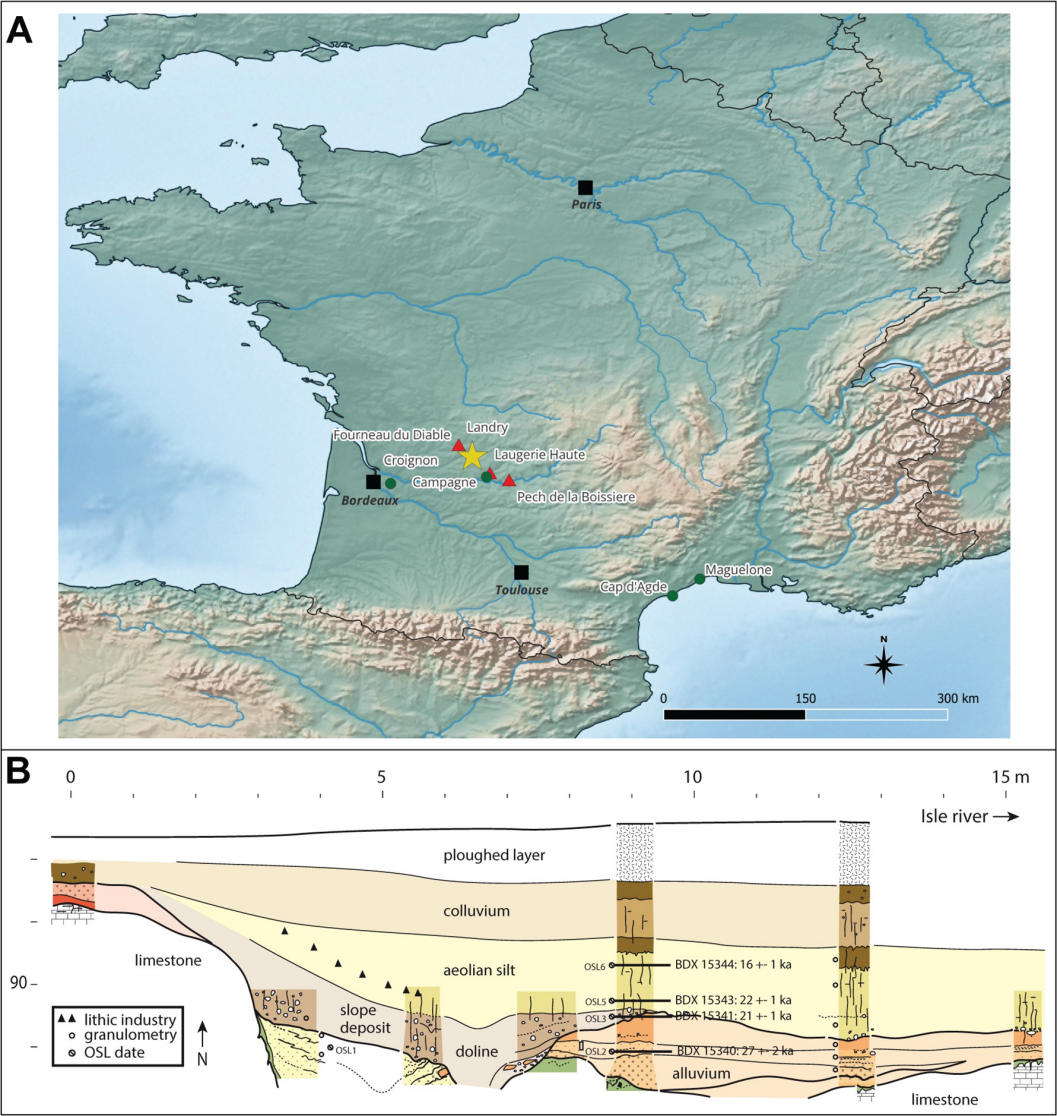
(A) Geographical map of France locating the Solutrean site of Landry in Dordogne (yellow star), the archaeological sites (red triangles) and the natural sites (green dots). (B) Synthetic stratigraphic profile and location of OSL dated samples to the west of the excavated area, according to Bertran (modified after [20]). The topography of the site slopes gently toward the Isle River and masks an alluvial terrace. Aeolian silts cover the layer on which the Solutrean hunter-gatherers settled. (A) Map created with QGIS 3.30.2 “s-Hertogenbosch”, background maps reprinted from Natural Earth. Free vector and raster map data @ naturalearthdata.com.

**Fig 3:**
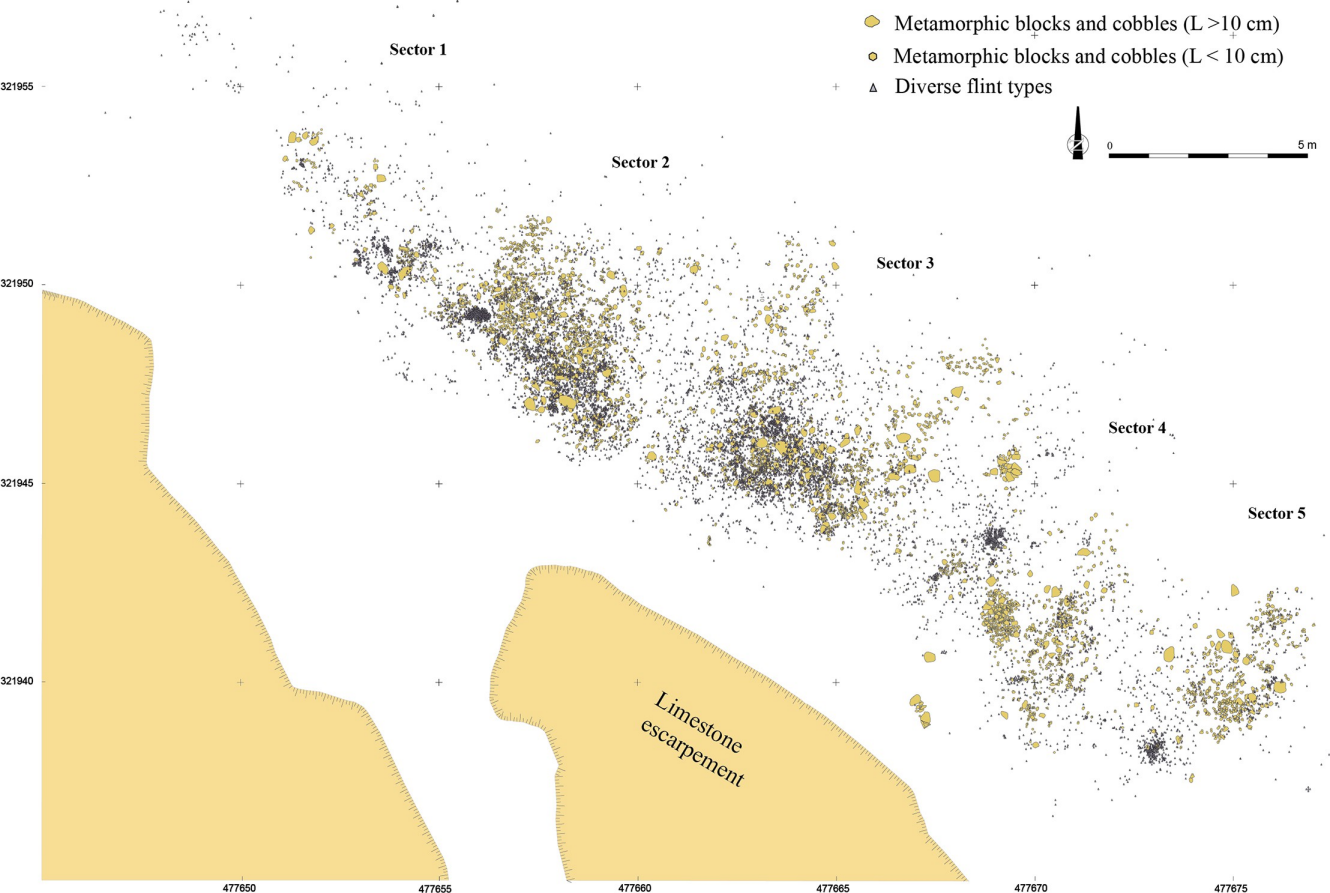
Spatial distribution of flint and metamorphic rock artifacts highlighting the five different activity sectors and size clusters [18].

MB excavated in 2011, Landry is an open-air site located close to Boulazac, Dordogne, France (Table 1). The site features several levels of aeolian silts overlying a low alluvial terrace of the Isle River (Fig 2). The Solutrean occupation layer is interstratified in the aeolian silts. Although periglacial processes, such as solifluxion, have slightly affected the uppermost layers of the site, geoarchaeological analyses [18] and refitting of stone artifacts show the well-preserved spatial distribution of archaeological finds has not been significantly disturbed (Fig 3). Furthermore, due to the well-drained soils, bacteria in an oxidizing environment have completely degraded organic matter such as bones. The remarkable diversity of the lithic raw materials used (local Senonian flint, and allochtonous flints, quartz, granite, and dolerite) and the discovery of schist slabs engraved with geometric patterns make this site unique in the region [19].

Spatial analysis identified five distinct concentrations of artefacts (Fig 3) spread over 300 m² in different excavated sectors, interpreted as resulting from different activities taking place during the same occupation [18]. This is particularly well evidenced by the analysis of sector 3. Combining data on the different nature, spatial distribution and use wear of artifacts, it is proposed that this concentration retains evidence of activities related to returning from hunting, flint tool knapping and shaping, butchery, skin preparation, hard organic material working, slab engraving and ochre processing [21, 22]. Thermoluminescence dating of burned flints has provided an average age of 21.5 ± 1.25 ka for the site [20], which is consistent with an attribution taking into account the margin of error, based on diagnostic stone tools found in the five concentrations, to the Late Solutrean. As many as 106 lustrous gravels were retrieved in the sieving from sector 3 and, to a lesser degree, from sector 2 (Figs 1 and 3). Those gravels are kept in the Inrap research laboratory of Campagne, Dordogne, France. All sediment from the excavated surface was sieved with a 2, 5 and 10mm mesh. The spatial origin of the gravels is known with a precision of a quarter of square meter.

### Fourneau du Diable

**Fig 4:**
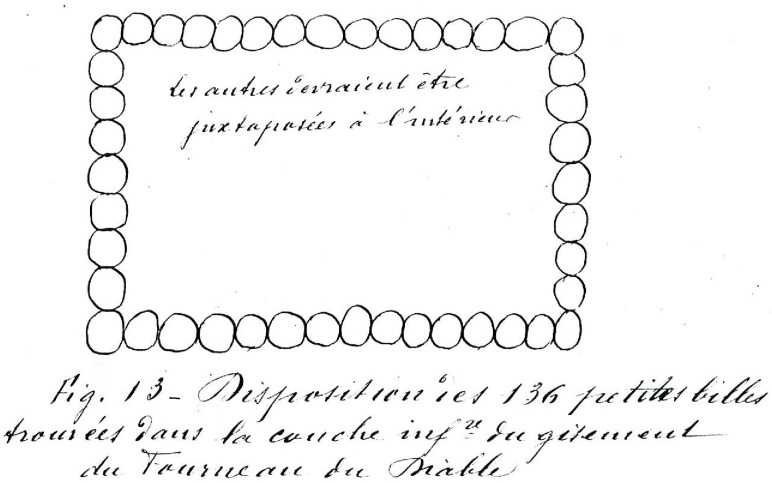
Drawing by Denis Peyrony of the gravels discovered at Fourneau du Diable [6]. Retranscription: « Les autres devraient être juxtaposées à l’intérieur. Fig 13 –Disposition des 136 petites billes trouvées dans la couche inférieure du gisement du Fourneau ». Translation: "The other 153 should be juxtaposed inside. Fig 13 - Arrangement of the 136 small balls found in the lower layer 154 of the Fourneau site".

This site is located on the right bank of the Dronne River, near Brantôme, Dordogne, France (Fig 2A). It consists of a small shelter, delimited to the north by a cliff, overhanging two superimposed terraces. The Quaternary sediments covering these terraces (Table 1) contains archaeological layers attributed to the Gravettian, the Solutrean and the Magdalenian [1]. These layers were covered by Holocene sediments and the fall of hanging blocks. Peyrony reports the discovery of lustrous gravels in the Gravettian or the Solutrean layers from the upper terrace [6]. According to this author, 136 spherical gravels, aligned and positioned closed to each other, were arranged in nine rows of 15 forming a rectangle (Fig 4). The pattern was interpreted by Peyrony as the remnant of a chest piece made of perishable material on which they were originally attached. These gravels are kept in the Musée Fernand des Moulins in Brantôme, Dordogne, France. New excavations, conducted by Baumann [1], led to the discovery of 465 new lustrous gravels (Fig 1). They were all recovered when sieving sediment from Gravettian and Solutrean layers. Forty gravels come from Gravettian layers and 16 from Solutrean layers, both located in the lower terrace. As many as 409 gravels were discovered in Late Solutrean layers located in the upper terrace. Gravels from this last excavation are curated in the Musée National de Préhistoire, Les-Eyzies-de-Tayac, Dordogne, France.

### Pech de la Boissière

Located near Carsac-Aillac, Dordogne, France (Fig 2A), the Pech de la Boissière rock shelter has a stratigraphic sequence comprising rich Solutrean and Magdalenian archaeological layers [7]. The archaeological deposit has suffered rock falls and runoff from the upper plateau. Of the 30 lustrous gravels discovered in the Solutrean layers by Peyrony, only nine are kept in the Musée National de Préhistoire, Les-Eyzies-de-Tayac (Fig 1 and Table 1).

### Casserole

One of the shelters overlooking the Musée National de Préhistoire, Les Eyzies-de-Tayac (Fig 2A), this comprises a stratigraphic sequence containing 13 archaeological layers attributed to the Gravettian, the Badegoulian, the Proto-Solutrean, the Solutrean and the Magdalenian (Table 1). Although geoarchaeological analysis shows that boulder falls, trampling, percolation, gelifraction and bioturbation have, albeit at small scale, contributed to the site formation process, the excavation conducted between 1991 and 1993 [3, 4], showed a low degree of disturbance of the Solutrean layers and identified a concentration of remains on a small surface interpreted as an area of intense human activity [3]. The Solutrean layers are dated by ^14^C AMS on burned bones to 20.5–23.3 cal BP [2, 23]. Fourteen lustrous gravels were retrieved in this layer (Fig 1 and Table 1). They are kept in the Musée National de Préhistoire at Les-Eyzies-de-Tayac.

### Laugerie Haute

Located at Les Eyzies-de-Tayac, on the right bank of the Vézère valley (Fig 2A), and firstly excavated in 1863 by Lartet and Christy, this iconic rock shelter has been since then the subject of numerous digs (Table 1). It includes two distinct sectors, Laugerie Haute East and Laugerie Haute West. The excavations yielded a long stratigraphic sequence encompassing Solutrean [24], in particular Proto Solutrean, Lower Solutrean, and Late Solutrean layers [25–27]. Radiocarbon dating indicates these layers were accumulated between 28 and 19 ka cal BP. Late Solutrean layers of Laugerie Haute West are dated by 14C AMS from 19 600 to 23 500 ka cal BP [28, 29]. Successive excavations conducted the last century by Peyrony and Bordes [25–27] at Laugerie Haute East have uncovered 19 lustrous gravels in layer 25 (Fig 1), attributed to the Late Solutrean. Eighteen gravels from Laugerie Haute West (Fig 1), of unknown stratigraphic origin, are also kept at the Musée National de Préhistoire, Les Eyzies-de-Tayac.

## Methods

For the sake of reproducibility and transparency, note that we use throughout the text the MeRIT system as per Nakagawa et al. [30]: initials of authors and acknowledged people are used to precisely identify individual contributions.

Table 2 below describes the various natural and geological factors that can modify the gravel surfaces. We thus rely on this inventory to better characterize the surfaces of archaeological gravels.

**Table 2:** Known natural processes producing luster on gravels.

	Wind erosion	Water	Ground or ice polishes	Chemical alterations	Animals
**Environments**	Desert, coast, periglacial region, mountains	River, sea	Periglacial region	Soil	Various (sea lions, galliformes, birds)
**Phenomena**	Abrasion	Transport (rolling, saltation, suspension), sedimentation	Solifluxion, cryoturbation, gelifluxion	Patina, corosion	Geophagy, attack of gastric juices, abrasion-polishing by organic or vegetable elements
**Macroscopic stigmata**	Shiny facets, sinuous ridges	Smoothed and shiny pebbles	Unworn shiny pebbles with accentuated reliefs	-	Gastroliths (small matted gravel)
**Microscopic stigmata**	Wide grooves with preferential orientation along the wind direction	V-shaped impact cupules, preferentially oriented microstriations, conchoidal fractures	Micro-polished, conchoidal fractures, parallel striations, circular grinding traces, impact cupules	-	Polished surfaces, microstriations

Data from [11, 31–49]

**Fig 5:**
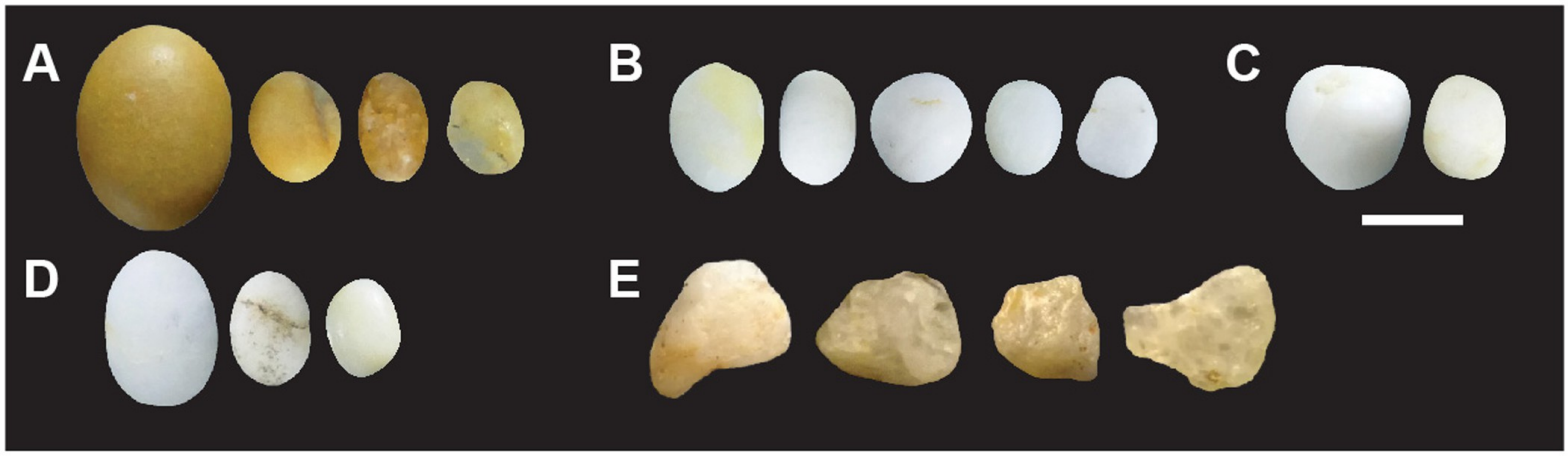
Examples of gravels from natural sites. (A) Gravels from the tertiary terraces of Campagne (Dordogne, France); (B) marine gravels from Maguelone (Hérault, France); (C) marine gravels from Cap d’Agde (Hérault, France); (D) gravels from the Quaternary alluvium and vineyards of Croignon (Gironde, France) and (E) gravels from the Landry’s sieving. These gravels were selected and collected according to previous observations of the Landry gravels and before having had knowledge of the lustrous gravels of the other archaeological sites whose raw material is visibly different. Scale = 1 cm.

Table 3 provides information on the studied material and the analytical techniques applied. This includes lustrous and non-lustrous gravels recovered at Landry, lustrous gravels from Fourneau du Diable, Casserole, Pech de la Boissière, Laugerie Haute, gravels from four natural deposits, gravels polished experimentally (see below), and the number of gravels presenting micro-concretions. Each archaeological gravel displays a similar degree of luster all over its surface. Permission was obtained to temporarily store and analyze the archaeological material at the PACEA laboratory, University of Bordeaux, Pessac, France. Lustrous gravels from Landry were retrieved in the sieving. Their white and shiny appearance facilitated their identification and comprehensive collection. Non-lustrous gravels come from Landry upper layers and were collected in the sieving from sectors bordering the area that yielded the lustrous gravels (Fig 5). LG and MB collected gravels from natural accumulations in 2018 (Figs 2A and 5). They come from several Mediterranean beaches located in the Hérault, dated from the Pliocene, Cap d’Agde and Maguelone beaches, Oligocene alluvial terraces located at Croignon, Gironde and Upper Cretaceous alluvial terraces close to Campagne, Dordogne.

**Table 3:** Number of gravels measured in the different approaches of this study for each archaeological sites including those collected in the sieve refusals, different natural environments and the experimental gravels.

		Concretions	Morphometry	Colorimetry	Surface texture analysis
	**Archaeological site**				
**Landry**		11	102	102	41
**Casserole**		1	14	14	4
**Fourneau du Diable**		288	465	465	11
**Pech de la Boissière**		2	9	9	3
**Laugerie Haute East**		2	19	19	5
**Laugerie Haute West**		3	18	18	5
	**Natural gravels**				
**Landry from sieving**			42	42	22
**Cap d’Agde**			2	2	2
**Maguelone**			5	5	5
**Campagne**			3	3	3
**Croignon**			3	3	3
	**Experimental gravels**				
**Process 1: gravels used on animal skin**			10	0	10
**Process 2: gravels used on ochred and greased animal skin**			10	0	10
**Process 3: gravels polished with silts from Landry**			2	0	2

Regarding the spatial analysis of the Landry gravels, we have included all the lustrous gravels in the analysis. Data on the spatial distributions of the gravels at the other sites were lacking, so we did not perform any spatial analysis.

### Distribution and spatial association of Landry’s lustrous gravels

It has been proposed that distributions of items at an archaeological site fall in three categories: concentration, uniform distribution, or random distribution [50, 51]. The occurrences of the items in a grid or their spatial coordinates can be used to establish to which of these three cases the distribution most closely approximates [52]. LG used gravels’ occurrences in the 50x50 cm Landry excavation grid to calculate the dispersion index with the help of FS. The *corrplot* package [53] was used to plot the spatial distribution of the gravels. The Poisson function law was applied to simulate numerous random distributions of the same number of lustrous gravels retrieved at the site (n = 106). The dispersion index was calculated and compared to simulated uniform distributions. A variance-to-mean ratio indicates whether the concentration of items is random or not.

LG explored the degree of spatial association between the lustrous gravels and major knapped stone tool classes, as well as between the former and artefacts made of metamorphic rocks, via a permutation test whose script was created by FS [54]. This test allows us to statistically verify the non-random association between two categories of artifacts by measuring their average Euclidian distance. This distance is then compared to values generated by a large number of runs in which the category of each object is randomly assigned, while the positions of all artefacts remain unchanged [55]. Since they were not spatially plotted during the excavation, we assigned Landry gravels random coordinates within their original 50x50 cm square. The results of this test give association indices for each pair of remains categories compared (gravel/flint, gravel/metamorphic rock).

### Experimental polishing

**Fig 6:**
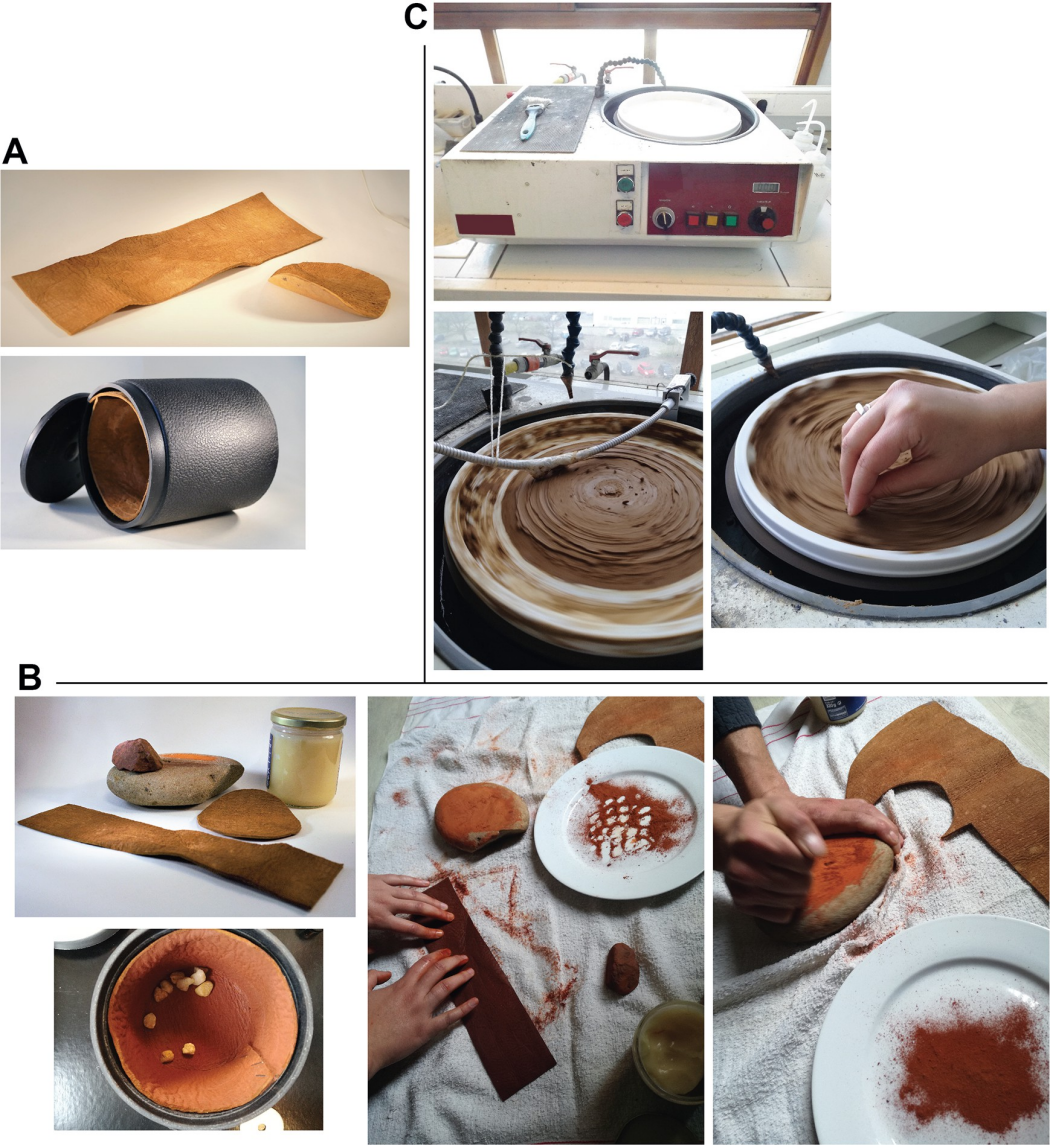
Presentation of the three experiments. (A) Material used for experiment 1, the animal skin used and the cylinder of the polishing drum. (B) Material used for experiment 2, the ochred and greased animal skin. Illustration of the *chaîne opératoire* reproducing an ochre tanning: obtaining the ochre powder and applying the ochre/grease paste on the hide. (C) Equipment used for experiment 3, the soil polish. A mechanical polisher covered with silt collected on the Landry site. Presentation of the manual (bottom left) then mechanical (bottom right) method of gravel gripping.

Three experiments were implemented to produce lustrous gravels. In the first two experiments LG used two models (3A and 33B/3-1.5) of rotary tumbler machines produced by Lortone Inc. (Mukilteo, WA, USA), commonly used to smooth and polish semiprecious stones. In the first two experiments, aimed at testing the effect of anthropogenic modifications, we used two animal hides. In the first experiment the inner surface of the barrel was covered with tanned cow leather (Fig 6A), in the second with a leather treated with a mixture of finely ground ochre powder and duck fat (Fig 6B). The choice of skin is justified by the fact that the available data suggest animal skin was widely used in the Gravettian and the Solutrean to produce articles of leather. As well, the small size of the gravels makes it unlikely that they could have been transported in nets or similar containers. Ten non-lustrous gravels from Landry were put in the tumbler barrel. The tumbler was set at 60 revolutions per minute and run for 504 hours. The gravels were cleaned under running water and acetone at the end of both experiments. The third experiment aimed at reproducing abrasion attested in periglacial environments, caused by sediments in contact with gravels mobilized by sedimentary processes [56–58]. A board covered with silt from the site of Landry, moistened with water, was attached to a metallographic polisher (ESC300 GTL model, produced by ESCIL, Chassieu, France) (Fig 6C). The gravels were manually held in contact with the silt-covered surface for 7 hours. They were then attached to a metal rod and maintained in contact with the silt for an additional 5 hours. Only one side per gravel was polished following this protocol. The polisher was operated at 110 rpm. The experimental gravels were then subjected to the same morphometric and roughness analyses applied to the other gravels analyzed (see below).

### Microscopic, morphometric and colorimetric analysis

Archaeological, experimental and natural gravels were photographed by LG and FdE with a Sony A6000 equipped with a Sony E30-mm F3.5 macro lens. Selected gravels were molded using Coltène^®^ President regular body high-resolution dental impression material and cast in resin (Epoxy Araldite 2020, Escil, Chassieu, France). LG and FdE observed and photographed the replicas in transmitted light at a magnification of 6-40x with a Leica Z6 APOA microscope equipped with a multifocus module. Additionally, the original archaeological, natural and experimental gravels were analyzed and photographed at a higher magnification of 200-400x using a Sensofar S-Neox confocal microscope, capable of generating in focus images similar to those obtained with a metallographic reflected light microscope. LG and AQ applied to digital images of archaeological lustrous and non-lustrous gravels as well as gravels collected at natural sites, the *Particle Analysis* plug-in of the image processing software ImageJ [59] to measure length, width, area, perimeter, and calculate circularity (4π*area/perimeter^2) and rounding indexes (4*area/(π*major axis^2). Thickness was measured manually using a digital caliper. LG and AQ extracted colorimetric values, expressed in RGB, by averaging the color of each gravel thanks to the Color Inspector 3D plug-in, and converted into L*a*b* with the *grDevices* package [60]. Inter-group comparison of the distribution of those quantitative data was performed through the Kruskal-Wallis non-parametric statistical test with the *pgirmess* package [61].

### Surface texture analysis

Confocal microscopy applied to archaeological artifacts is able to distinguish wear signatures of natural and anthropogenic agents [10, 11, 17, 62]. LG analyzed archaeological lustrous and non-lustrous gravels from Landry and lustrous gravels from Fourneau du Diable, Casserole, Pech de la Boissière, Laugerie Haute, from natural deposits and gravels experimentally polished (Table 3) with a Sensofar S-Neox confocal microscope equipped with a long-working-distance 50x objective (numerical aperture = 0.45), allowing a spatial sampling of 0.26μm. We measured on opposite aspects of each gravel two areas of 351x264μm with a vertical repeatability of 3 nm and 42° of maximum acquisition slope. Measures were taken on areas free of micro-concretions. LG and AQ processed the data obtained with the SensoMAP Premium 7.4 software. Post-acquisition data treatment followed a procedure similar to that described by Martisius and colleagues [63]. Using built-in operators, this included levelling the surface with the least squares method, removing isolated outliers and noise, filling in non-measured points by interpolating from neighbours, and removing the surface form using a polynomial of third order.

A Gaussian filter of 80μm was applied as a cut-off to remove waviness from roughness, which appeared to separate roughness values of glossy from non-glossy gravels better than the 25μm filter. Three ISO 25178 parameters were calculated (Table 4): the root mean square height (Sq*)*, the degree of bias of the shape of the roughness (Ssk), and the developed surface ratio (Sdr). Previous studies have shown the pertinence of these parameters to characterize different degrees of polish on archaeological artefacts [11, 39, 64]. In addition, they present the advantage of being largely independent from one another. Univariate and multivariate statistical analyses were applied to these data. We present here the results of two PCA (Principal Component Analysis) with convex hulls for each group of gravels. We obtained those PCA via the FactoMineR package [65].

**Table 4:** Three ISO 25178 parameters used to measure the textural surfaces.

**Amplitude parameters (characterization of the z axis perpendicular to the surface)**		
**Sq**	**μm**	Root mean square height, standard deviation of heights. A low roughness will have a Sq value equal to 0 while a high roughness will have a Sq value superior to 0.
**Ssk**		Skewness. Degree of bias of the shape of the roughness (asperity). A low roughness will have a Ssk value under 0 (surface dominated by valleys) while for high roughness le Ssk value will have a value superior to 0 (surface dominated by peaks).
**Hybrid parameters (information present on the three axes x, y and z of the surface)**		
**Sdr**	**%**	Developed surface ratio. 0% indicates a flat and smooth surface, 100% indicates a complex surface with large peaks and valleys.

We selected two amplitudes parameters: the root mean square height (Sq), the degree of bias of the shape of the roughness (Ssk), and one hybrid parameter: the developed surface ratio (Sdr).

### Ethnographic survey of small pebble use

To explore historical and contemporary uses of small stones similar to the lustrous gravels examined, FMJ used the electronic Human Relations Area Files (eHRAF), a collection of full-text, subject-coded global ethnographic materials (http://www.ehrafworldcultures.yale.edu/). A text search on [“pebble* OR stone*” NOT subject:building and construction] was performed (the term “gravel*” yielded only 15 descriptions of material used for paths). The search was restricted to the 186 Standard Cross Cultural Sample (SCCS) cultures, a subset of eHRAF with the most detailed and robust ethnographic descriptions. This yielded 23022 paragraphs across 175 cultures. The eHRAF Outline of Cultural Materials (OCM) has subject codes tagged to each paragraph. The top five for this search were Sacred Objects and Places, Prayers and Sacrifices; Mythology; Literary Texts; Spirits and Gods. Utensils, Tools, Weapons, and Burial Practices were in the top 20. Because each paragraph is usually tagged with multiple OCM codes, we developed a bespoke coding scheme for specificity.

All material in a sample of 82 cultures (all 38 hunter-gatherer societies in the SCCS plus 44 others stratified by region, c.20% in each totalling 10488 paragraphs) was read and coded for how pebbles were used. FMJ noted if pebbles were present in multiples of four or more at one time (Yes, No, Unclear), and any information about bags or other carrying items. We did not code keyword mentions that were irrelevant to our study, especially where stones were described as larger than a fist (e.g. grinding or cooking stones, cairns, commercial use) or when the keywords were associated with technologies not relevant for our study (e.g. pebbles associated with metal or glass in ornaments, pebbles with holes for stringing, for use as weights in commercial trade etc.). Other rare mentions, such as being described in fantastical folklore, or stones referring to the seeds of fruit, were also not assigned to a code.

## Results

### Distribution and spatial association of lustrous gravels at Landry

**Fig 7:**
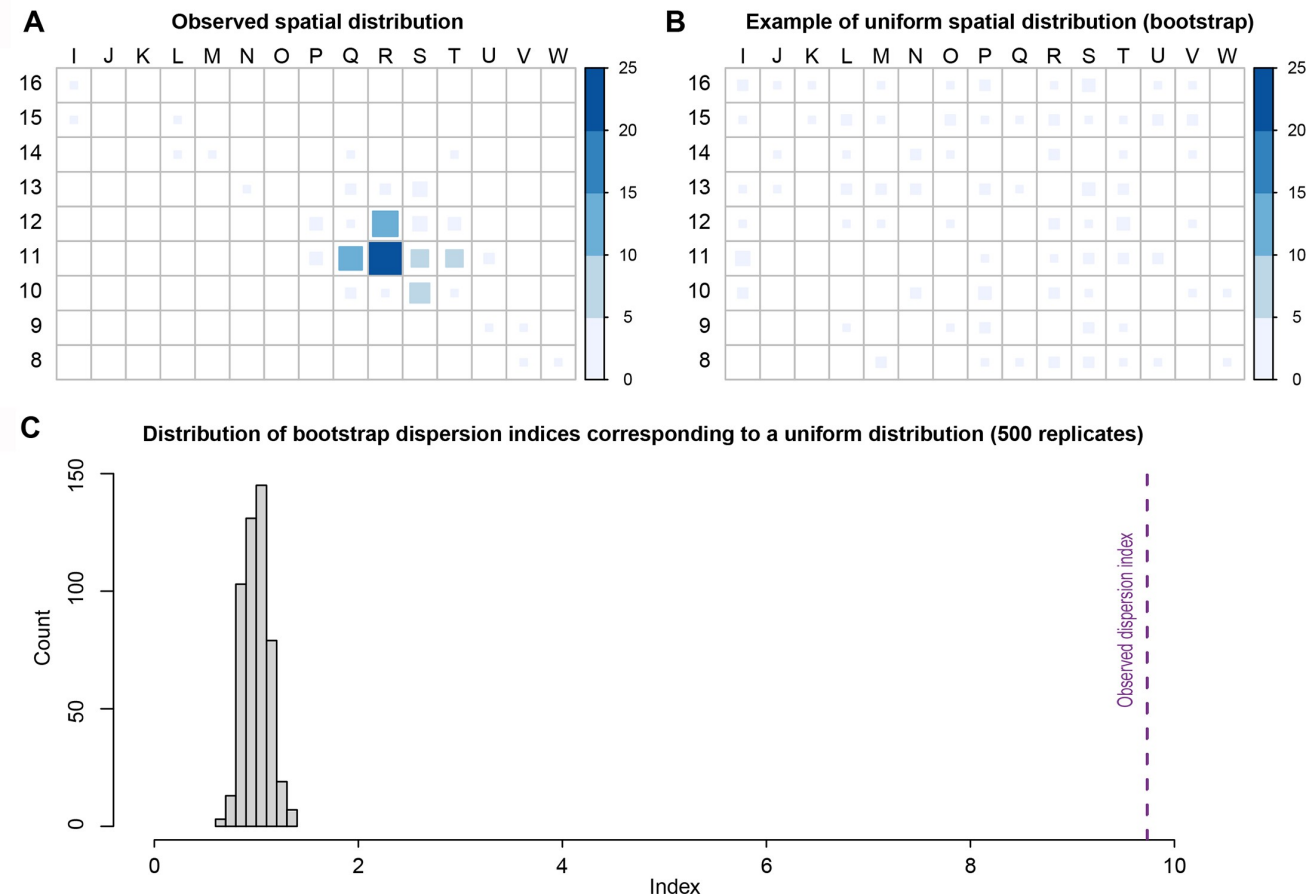
Graphics presenting the results of the spatial analysis. (A) Count of gravels in the Landry grid. This area corresponds to sector 1 (squares columns I through J), sector 2 (columns K through P), and sector 3 (columns P through V). (B) Simulation of a uniform spatial distribution of gravel on sector 1, 2 and 3. (C) Distribution of dispersion indices corresponding to a uniform distribution (500 random draws) and on the right in purple, dispersion index associated with real glossy gravel’s distribution.

Lustrous gravels are concentrated in squares R11, R12, and Q11, sector 3, and to a lesser extent in adjacent squares (Fig 7). A few gravels are present in sector 2. They are virtually absent in the rest of the excavated area. This is confirmed by a dispersion index of 9.7, much higher than that expected for a uniform or random distributions (Fig 7).

**Fig 8:**
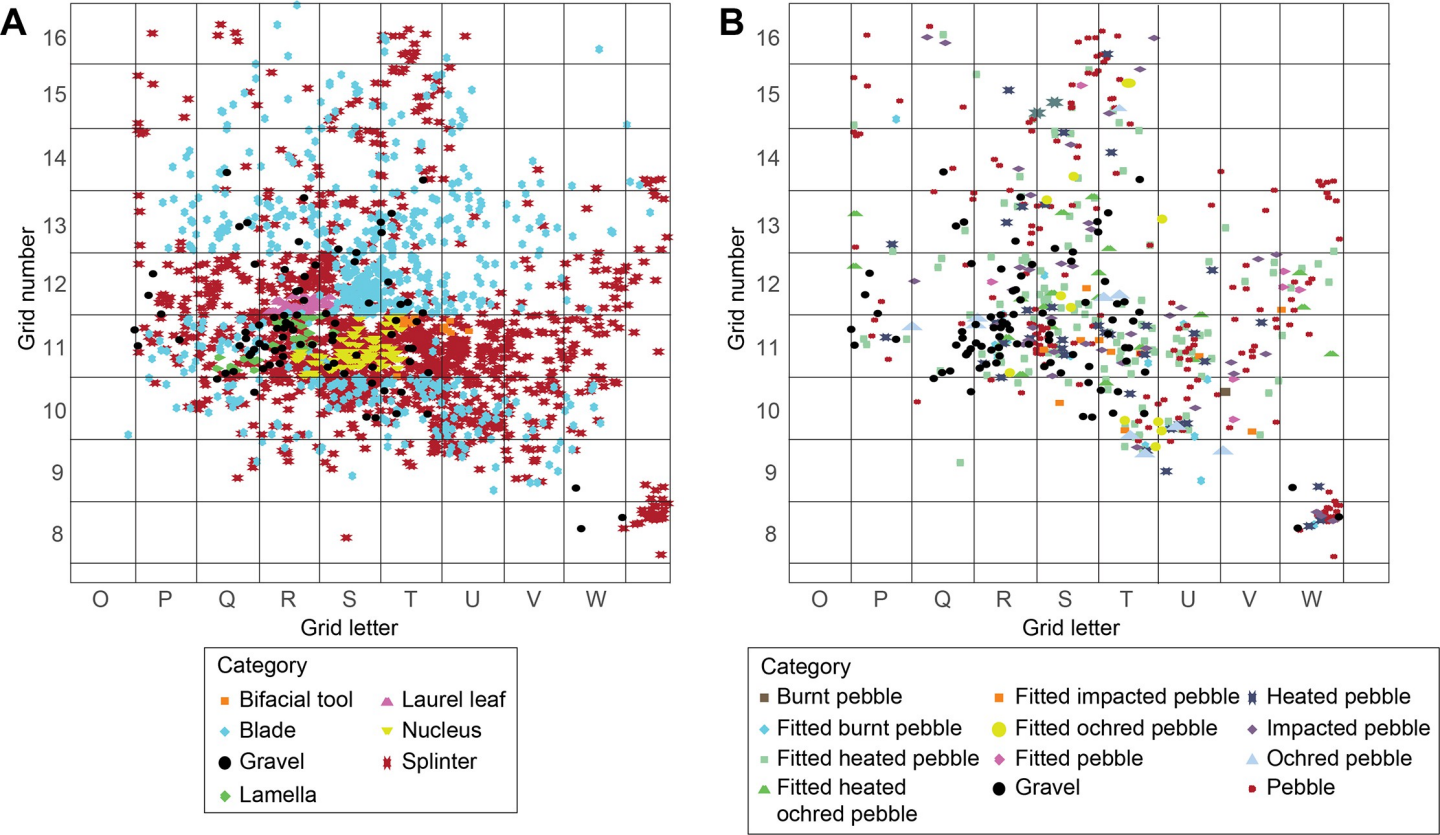
(A) Spatial distribution observed on sector 3 of different flints tools and lustrous gravels and (B) of the latter and metamorphic stone tools.

**Fig 9:**
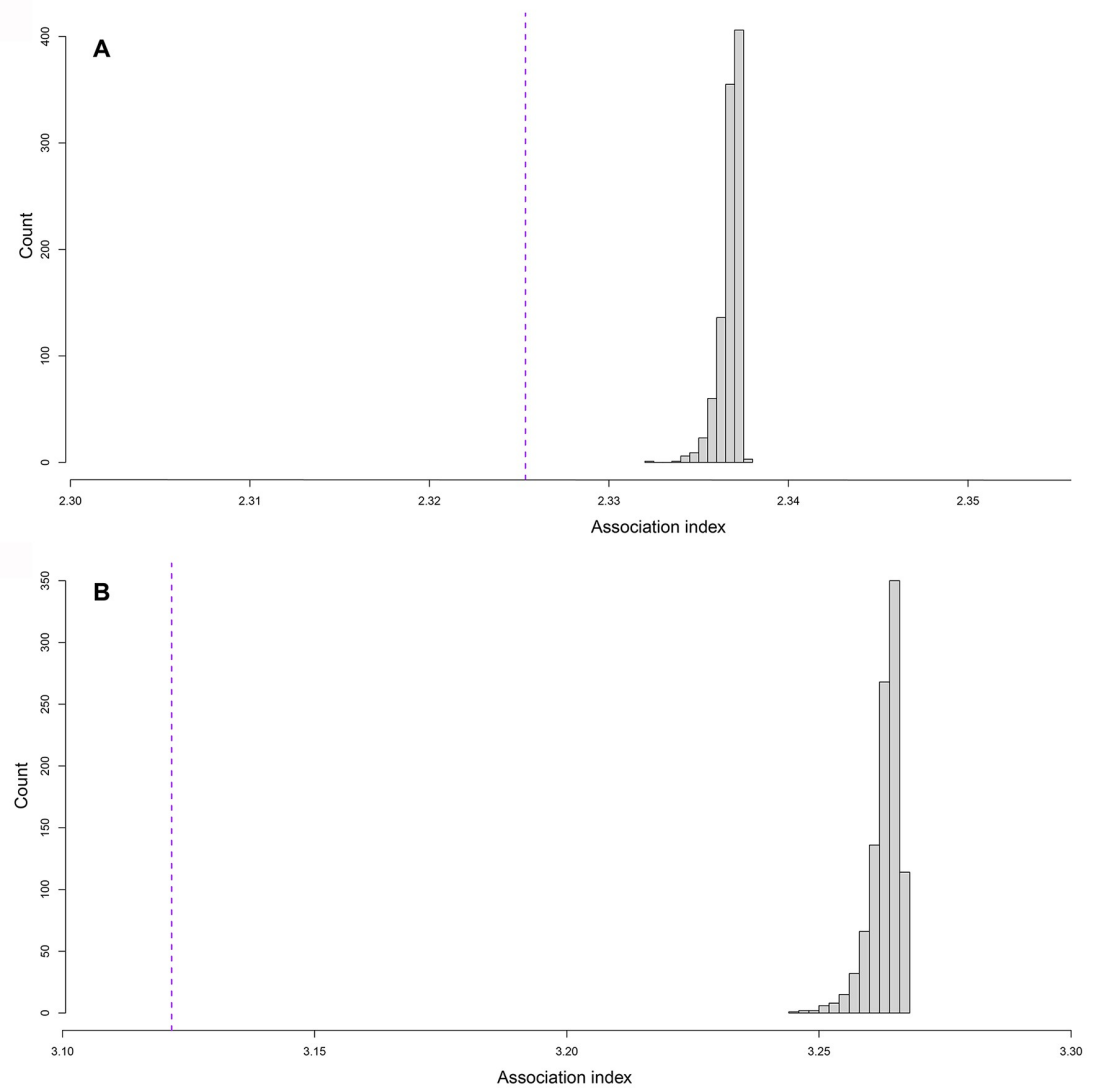
Permutation tests displaying association indices. (A) The dashed line shows the association index between gravels and flints and (B) metamorphic rocks. Histograms represent the distribution of indices over 500 draws with random position permutations.

The spatial distribution of flints and metamorphic rocks appears strongly correlated with that of the lustrous gravels (Fig 8). As indicated by a significant difference in association indices (Fig 9), the average distance between the gravels and the objects in the other categories is greater than if the category had been randomly assigned to objects with the same geographic coordinates. This result establishes that the concentration of gravels is stronger than that of the other categories of remains.

### Morphometry and colorimetry

**Fig 10:**
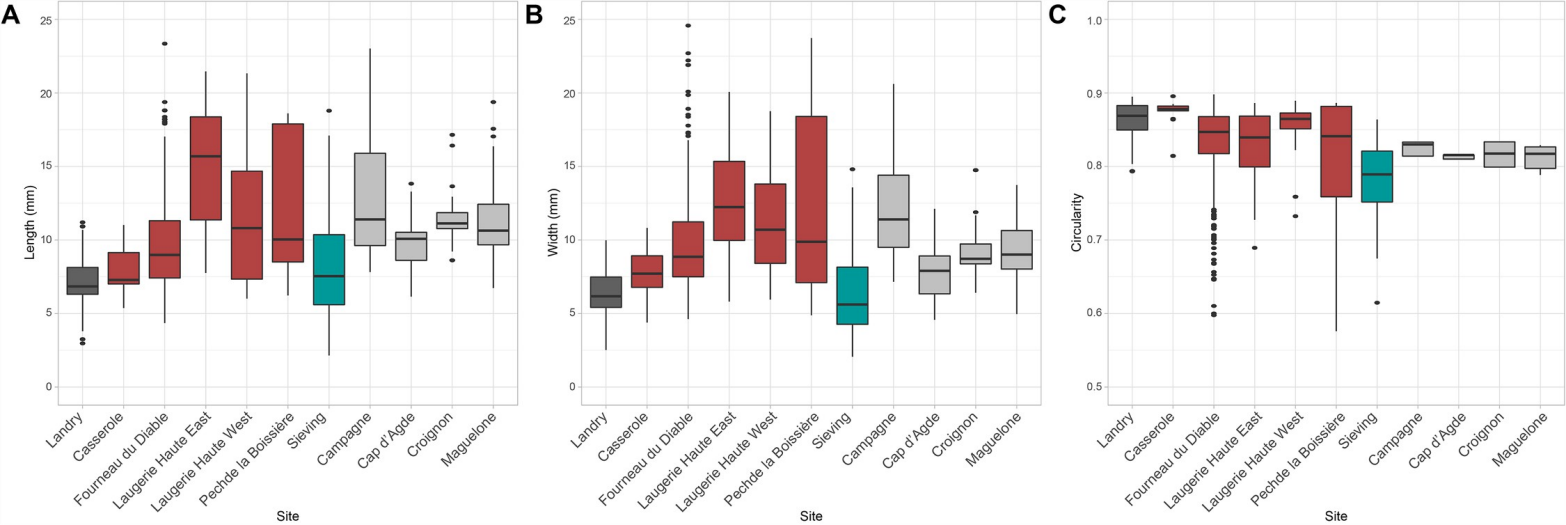
(A) Comparison of gravel length, (B) width and (C) circularity index for each site. The circularity index is close to 1 for a round object and 0 for an object of more complex morphology.

**Fig 11:**
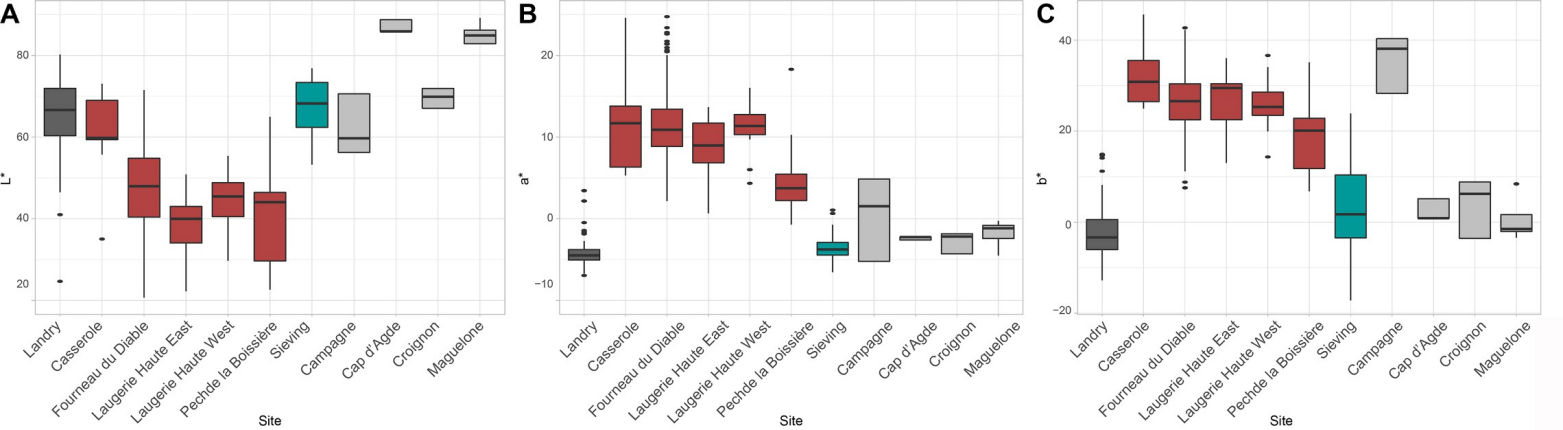
Representation of gravel color according to the parameters L* (luminescence, 0 = black, 100 = white), a* (green-red axis) and b* (blue-yellow axis). The two parameters range from +127 to -128.

Morphometric analysis identifies significant differences (p < 0.05) (see S1 Table) between lustrous gravels found at Landry, Casserole, and a part of those from Fourneau du Diable, which are smaller and more spherical (Fig 10). These are also different from those at Pech de la Boissière, Laugerie Haute, a part of those from Fourneau du Diable, those of Landry retrieved outside the concentrations and those from natural sites, which are more elongated and more variable in size. The gravels from the Fourneau du Diable, Pech de la Boissière and Laugerie Haute sites are also more homogenous in color; less so for the others (Fig 11 and S1 Table). At Landry no significant differences are observed in the color of lustrous and not-lustrous gravels, since they are all made of quartz.

### Microscopic analysis

**Fig 12:**
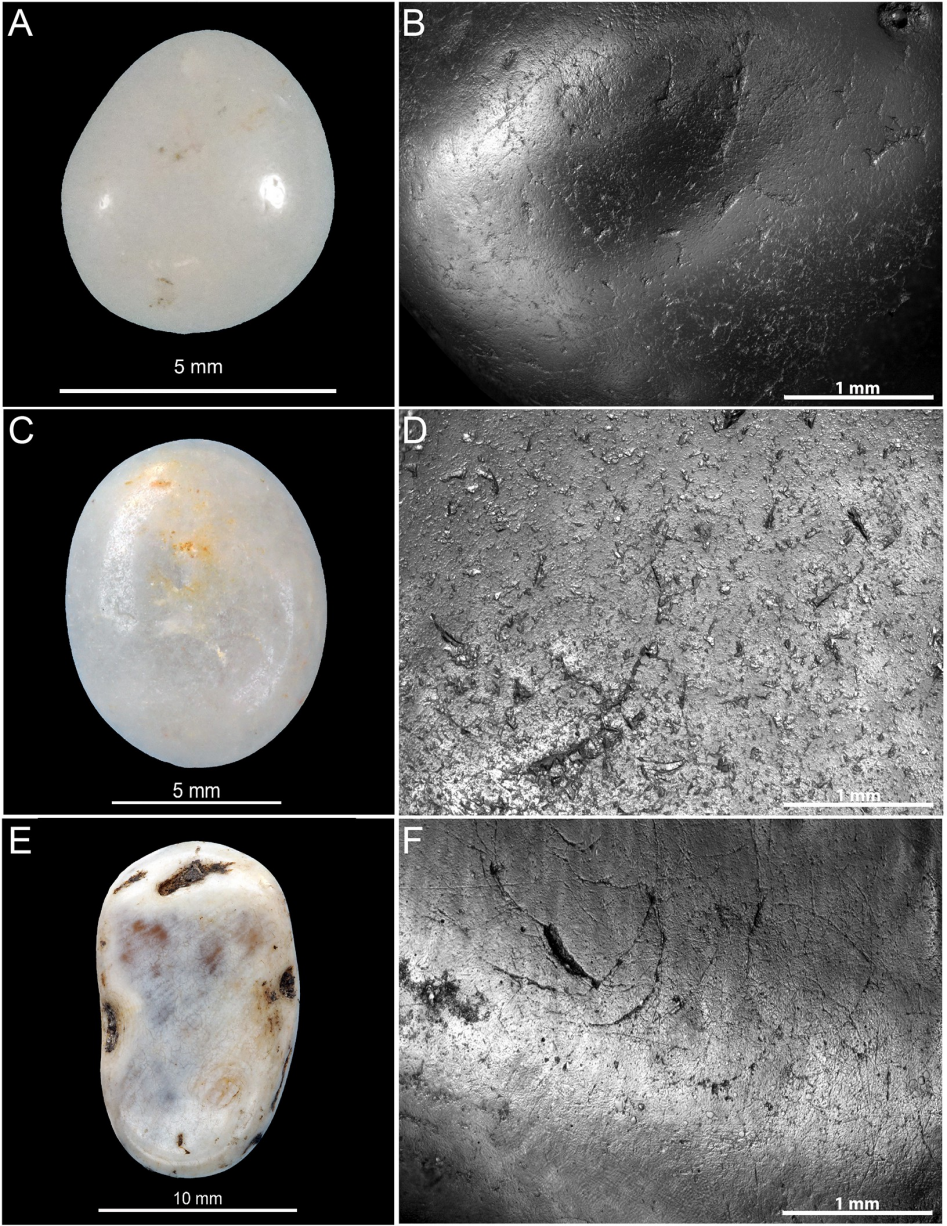
Macroscopic (left) and microscopic (right) photos of gravels from archaeological sites illustrating the luster on surfaces. (A-B) Gravel n°R12D_64 from Landry; (C-D) gravel n°K11D50_5 from Casserole and (E-F) gravel n°109b from Fourneau du Diable.

**Fig 13:**
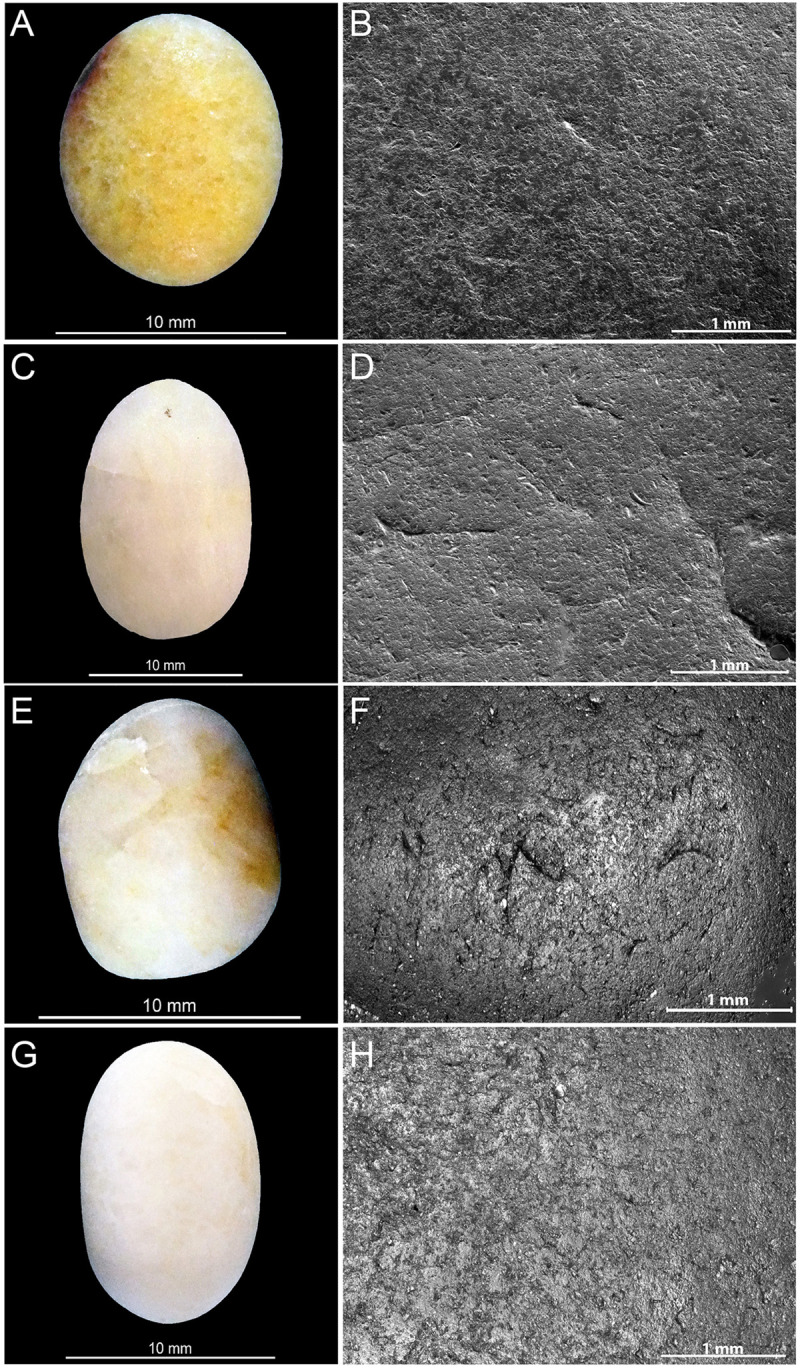
Macroscopic (left) and microscopic (right) photos of gravels from natural sites illustrating the variability of surfaces. (A-B) Gravel n°627 from Campagne; (C-D) gravel n°628 from Croignon; (E-F) gravel n°654 from Cap d’Agde and (G-H) gravel n°688 from Maguelone.

**Fig 14:**
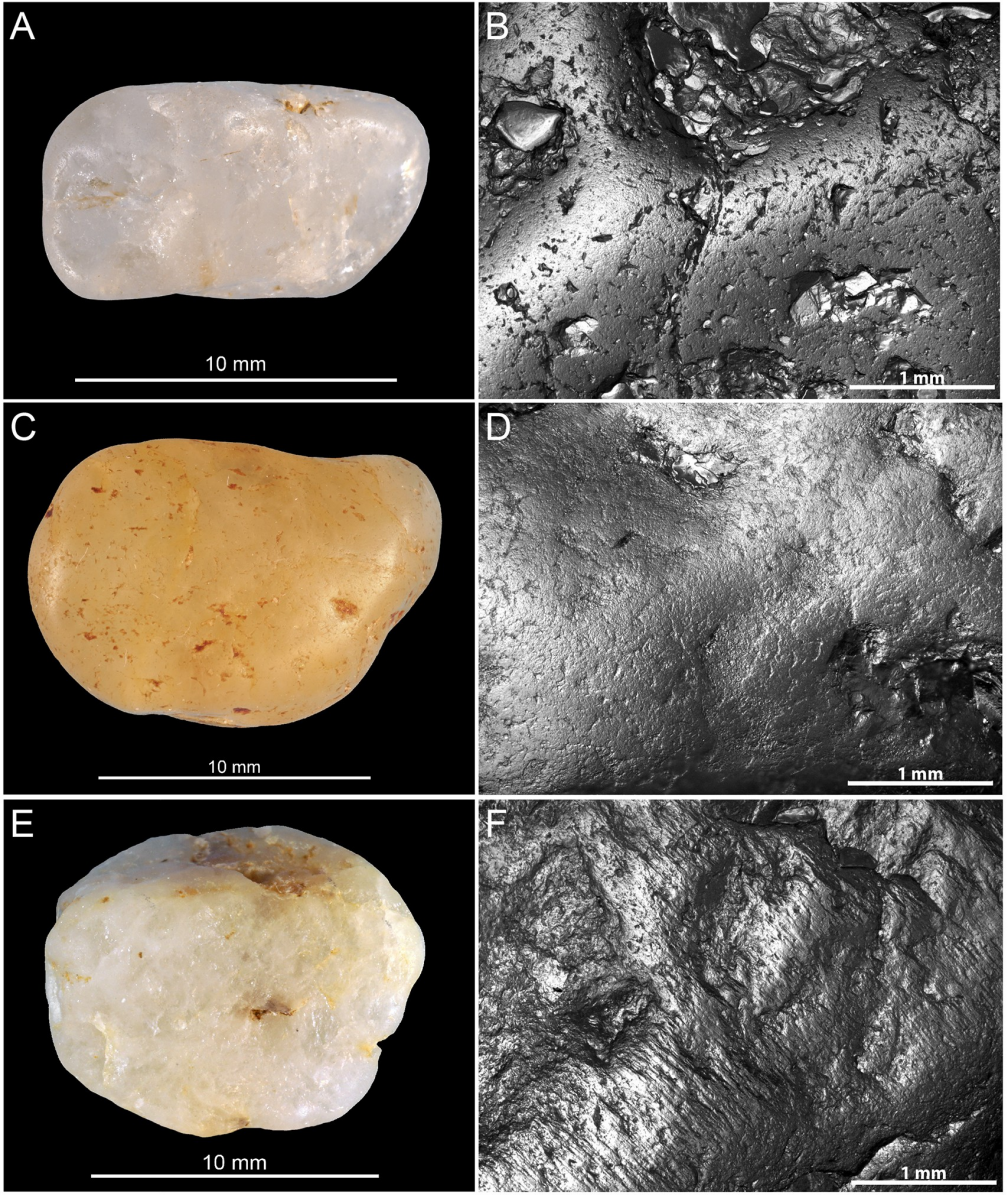
Macroscopic (left) and microscopic (right) photos of experimental gravels. (A-B) Gravel n°313 from the experiment processing the animal skin; (C-D) gravel n° 129 from the experiment processing the ochred skin with animal fat and (E-F) gravel n°137 from experiment processing wet silt from Landry.

The archaeological lustrous gravels are characterized by homogeneous surfaces on which depressions are either absent, or rare and of dimensions not exceeding 10μm. Curved micro-cracks corresponding to incipient fracture cones are also discernable (Fig 12). The edges of these fracture lines occasionally reveal chipping and conchoidal micro removals. The frequency of depressions and the degree of surface homogeneity is dependent on the degree of luster observed to the naked eye. The natural gravels (Fig 13) which are visually matte are characterized at the microscopic scale by more uneven surfaces than those observed on the archaeological pieces. The natural gravels present small homogenous areas exclusively on prominent zones. This is particularly evident on pieces from river terrace deposits (Fig 13A and 13B). Gravels collected on beaches (Fig 13C and 13D) show more intense pitting associated, in some cases, with incipient fracture cones not flattened by wear, as is the case for archaeological gravels. The surfaces of gravels tumbled in barrels are similar to those on archaeological specimens without reaching the same level of homogeneity (Fig 14). Notably, treatment with tumblers covered with ochred skin (Fig 14A and 14B) produces the surface modification comparable to those observed on archaeological gravels. Abrasing natural gravels with a polishing machine covered with wet silt produces lustrous areas entirely covered with 20–50μm wide parallel striations (Fig 14C).

**Fig 15:**
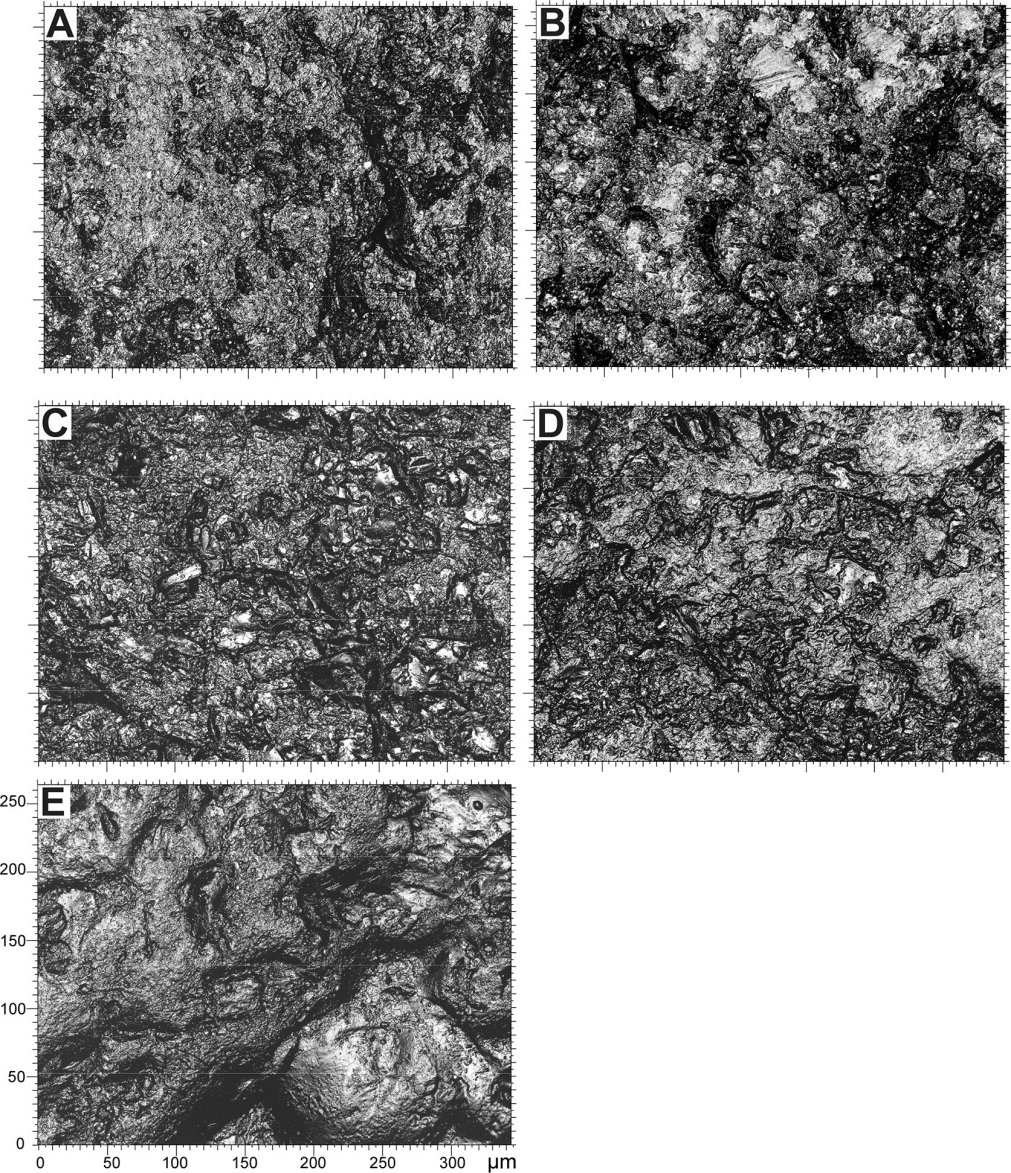
Micrographs of natural gravels. (A) Gravel n°653 from Cap d’Agde and (B) gravel n°684 from Maguelone marine beaches; (C) gravel n°628 from Croignon and (D) gravel n°627 from Campagne alluvial terraces, and (E) gravel n°170 recovered in the sieving at Landry.

**Fig 16:**
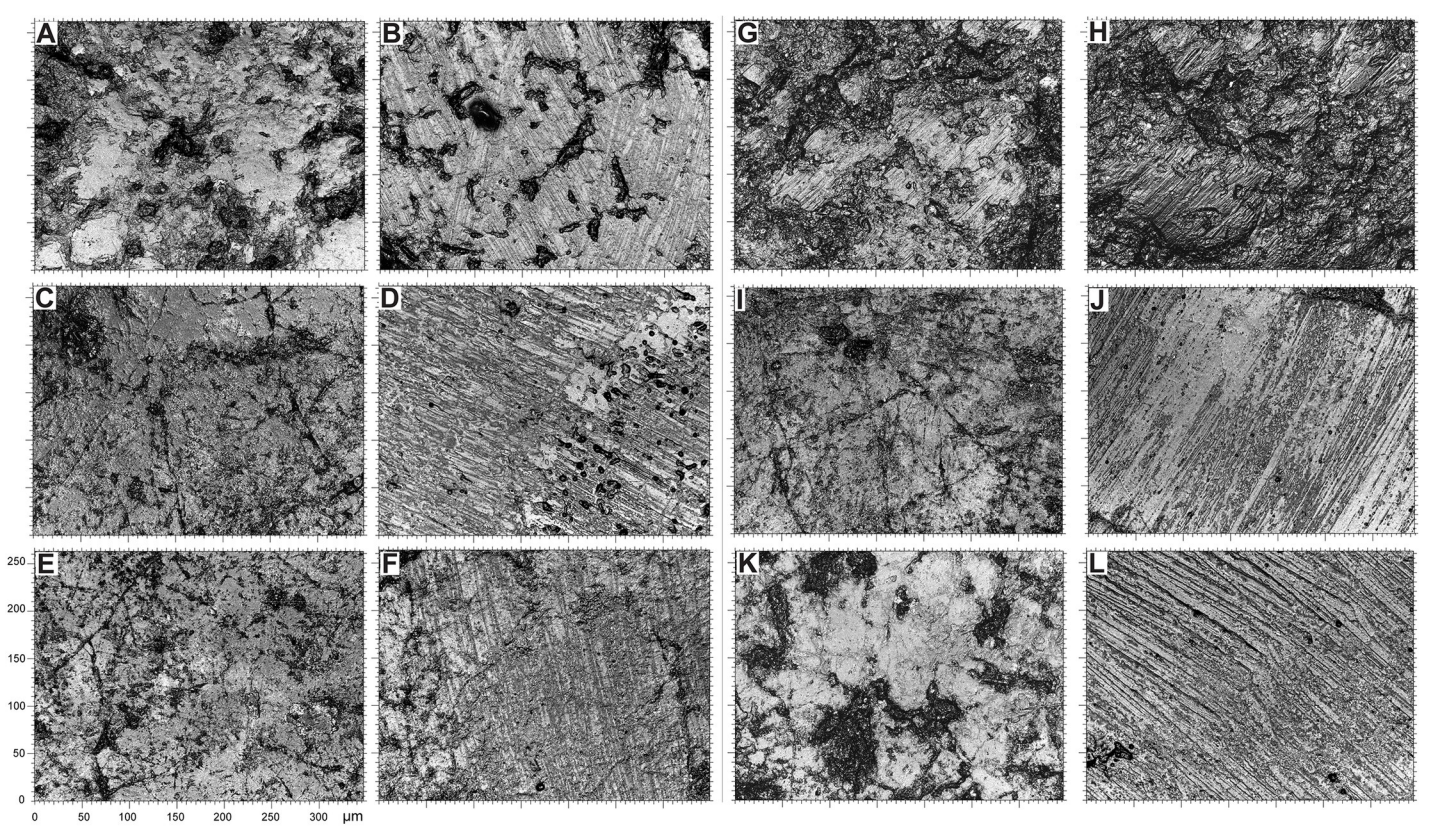
Micrographs of archaeological gravels. (A-B) Gravels n°P12A_10 and n°Q11C_18 from Landry; (C-D) gravels n°3_1192 and n°4_1193 from Casserole; (E-F) gravels n°459b_1183 and n°443b_1167 from Fourneau du Diable; (G-H) gravel n°511b_1249 from Pech de la Boissière; (I-J) gravels n°470b_1228 and n°501b_1239 from Laugerie Haute East and (K-L) gravels n°469b_1207 and n°472b_1210 from Laugerie Haute West.

At higher magnification, the surface of all natural gravels and those recovered in the sieving at Landry retain a rough appearance associated with micro concavities ranging from 20 to 100 μm in length (Fig 15). Rare lustrous areas, not exceeding 20 and 50 μm in length, are observed on gravels from marine beaches and alluvial terraces respectively. High magnification analysis of archaeological gravels reveals a substantially different and more diversified wear pattern. All examined collections include gravels displaying either large lustrous surfaces resulting from the flattening of prominent areas, with few concavities and no or few randomly oriented striations, (Fig 16A, 16C, 16E, 16G, 16I and 16K) or surfaces characterized by a complete or almost complete flattening of the gravel surface associated with parallel or subparallel 2–5 μm wide striations (Fig 16B, 16D, 16F, 16H, 16J and 16L). The two wear patterns are associated on some gravels. Intriguingly, striated areas exhibit either individual overlapping striations (Fig 16J and 16L) or bands of parallel striations indicating a wider contact with the abrasive material. Moreover, in specific cases (Fig 16D, 16J and 16L), the areas covered with striations display signs of additional polishing, leading to the flattening of prominent regions between striations (Fig 16D and 16L) or their occasional disappearance (Fig 16J).

### Surface texture analysis

**Fig 17:**
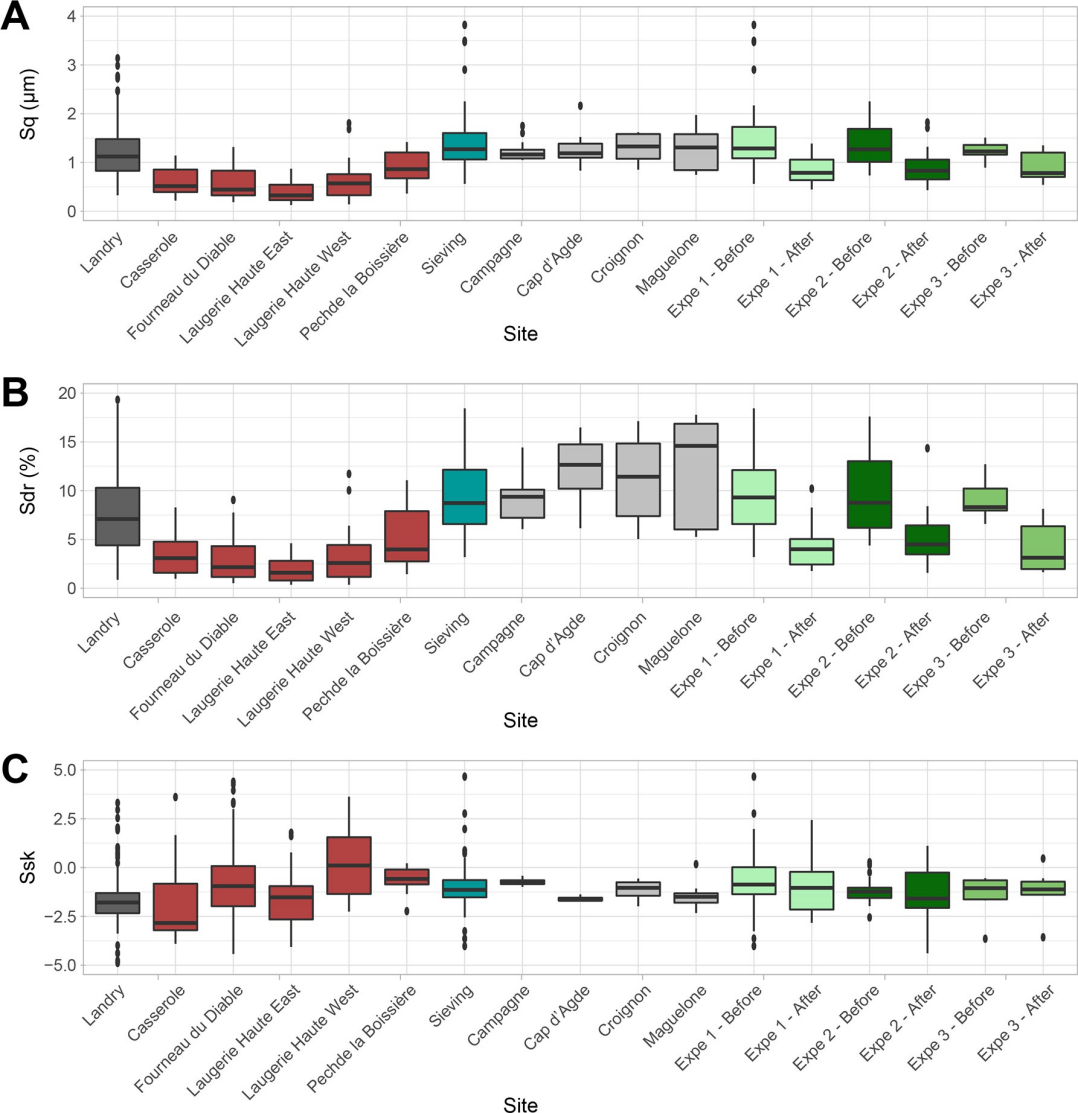
(A) The distribution of Sq (μm) parameter, (B) the distribution of the Sdr (%) parameter and (C) the distribution of Ssk parameter.

**Fig 18:**
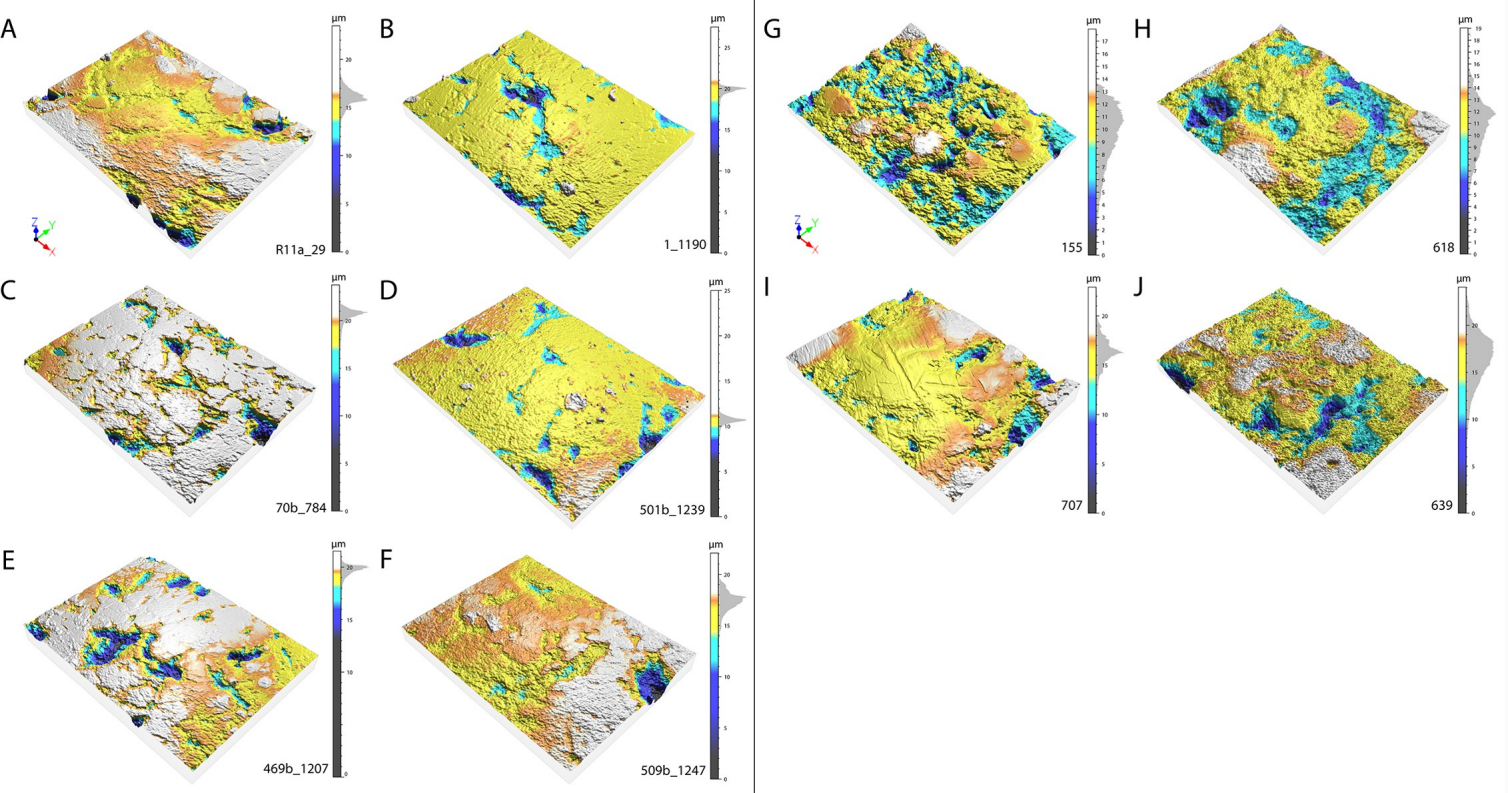
Three-dimensional representations of the surfaces of four gravels from different sites. The Landry gravel (A) has a Sq of 0.98μm and a Sdr of 4.45%, its surface is of intermediate roughness. The Casserole gravel (B) is quiet similar to Landry one. The Fourneau du Diable gravel (C) has a Sq of 0.84μm and a Sdr of 4.98%, it is characterized by a regular luster. The Laugerie Haute East gravel (D), Laugerie Haute West gravel (E) and Pech de la Boissière gravel (F) also present a regular luster. The gravel from the Landry’s sieves (G) has a Sq at 1.69μm and Sdr at 16.4% and the gravel from Croignon (J) shows a Sq values at 1.75μm and a Sdr at 16%. They are both characterized by a rough surface. The Campagne gravel (H) and Maguelone gravel (I) present similar values than (G) and (J) and their surfaces are rougher. These visualizations highlight the folds of the glossy surfaces as well as the shape of the histograms on the Z-scales expressing the distribution of the points with respect to the average plane of the surface.

Roughness parameters identify significant differences (p < 0.05) (see S2 Table) between natural and most archaeological gravels (Fig 17). Gravels from Casserole, Fourneau du Diable and Laugerie Haute show low Sq and Sdr values, which reflect smooth and not very complex surfaces (Fig 18). The positive reliefs of the micro-topography appear erased, leaving a flat surface with hollows and valleys (Fig 18C). This wear pattern gives these gravels a strong shine. In contrast, the gravels from the Landry sieving and from natural sites have rougher surfaces, resulting in a dull appearance. The lustrous gravels from Landry are significantly rougher than those found in the other archaeological sites (p < 0.05) (see S2 Table). They are, on the contrary, not significantly smoother (p > 0.05) (see S2 Table) than those from natural sites but significantly smoother than those recovered at Landry outside the archaeological concentrations. This intermediate state of surface for the Landry lustrous gravels comes from more heterogeneous surface textures: some individuals show values close to those recorded on gravels from the other archaeological sites, and some show values close to those from the Landry sieving and natural sites. In summary, the gravels from archaeological sites and lustrous Landry gravels display a roughness incompatible with that recorded on gravels from natural sites.

### Multivariate analysis

**Fig 19:**
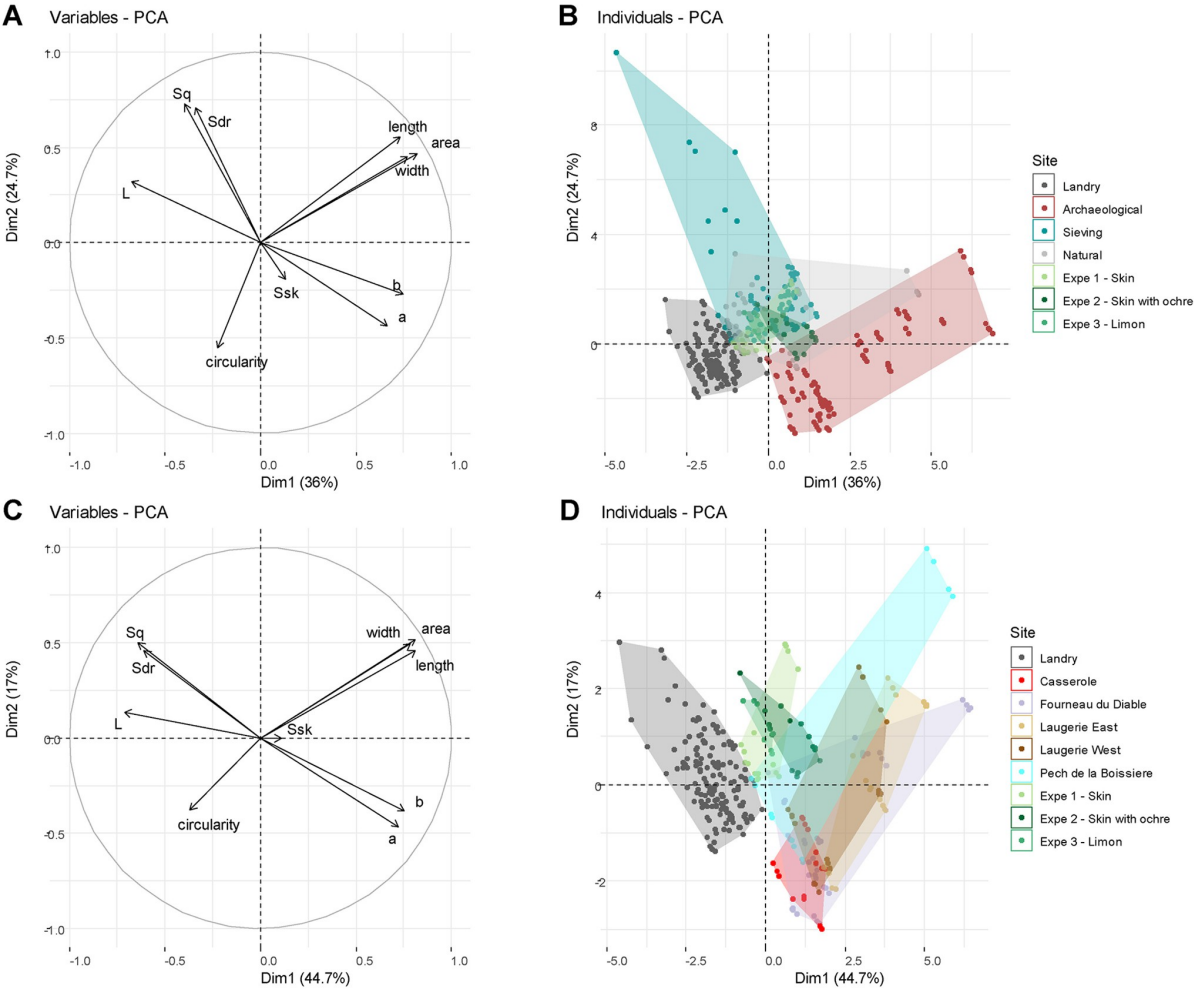
Principal component analyses taking into account morphometric, roughness and colorimetric variables. The PCA in (A) and (B) compares natural, experimental and archaeological gravels. Gravels from Landry and those retrieved during the sieving of the sediments from this site are plotted separately. The PCA in (C) and (D) compares experimental gravels with those found at each archaeological sites. Dots correspond to one of the four measurements performed on each gravel: 35 natural, 69 archaeological and 22 experimental gravels.

The principal component analysis of all recorded parameters (Fig 19) highlights a clear difference between gravels found at archaeological sites excavated in the past, natural gravels and gravels found at Landry both inside and outside the concentrations of archaeological artifacts. While very few gravels from the Landry concentrations display features compatible with those recorded on natural populations, a consistent portion of those found outside the concentrations are comparable with the natural ones. A clear difference appears also between the lustrous gravels of Landry and those from the other archaeological sites. The former are more circular, whiter in color, and display comparatively rougher surfaces.

### Experimental results

**Fig 20:**
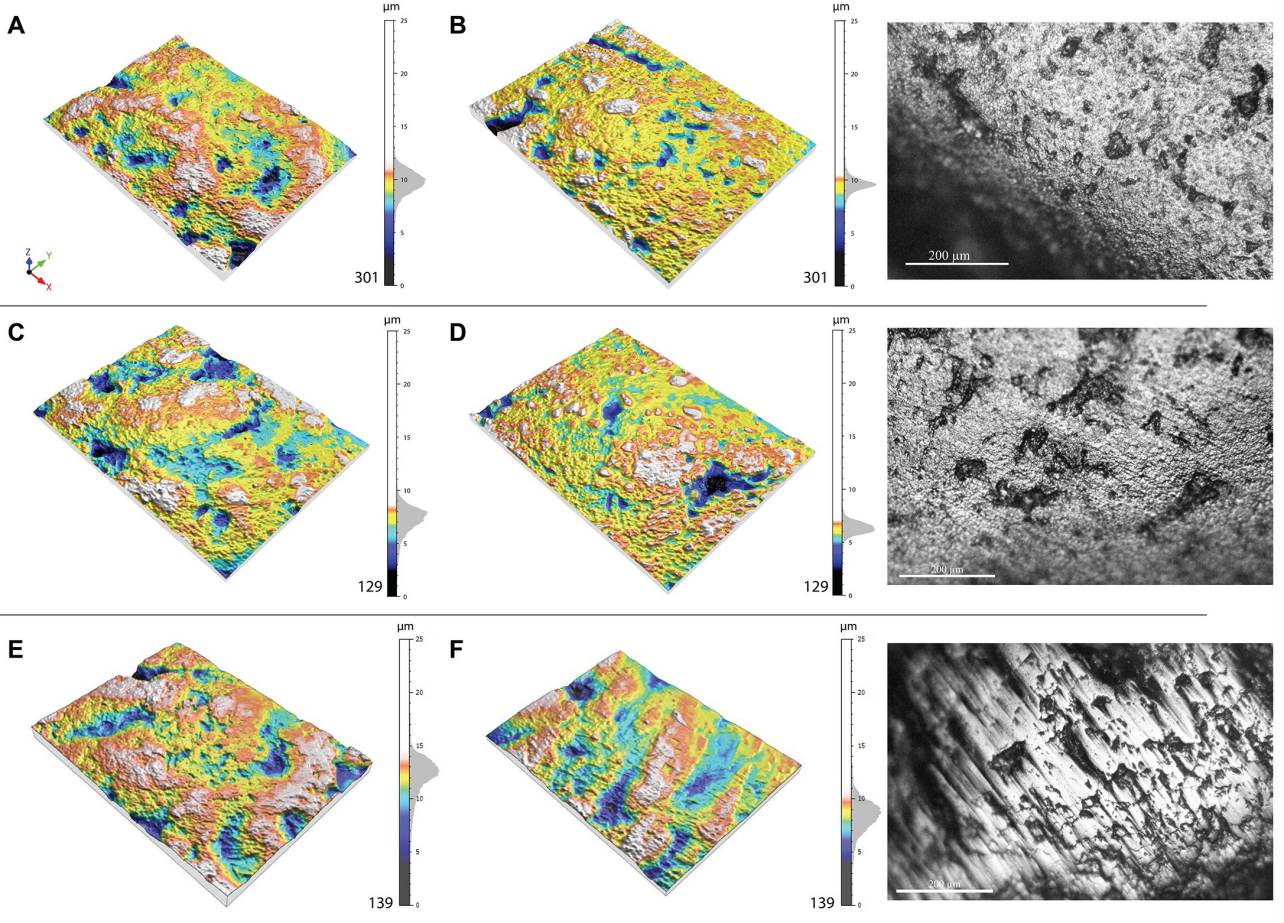
Three-dimensional visualization of the surfaces before and after experimentation. Process 1 (gravels used on animal skin) has erased the reliefs in favor of hollows and valleys and the polish is more homogeneous. (A) Shows the 3D surface of the experimental gravel before the experiment and (B) shows the surface of the same gravel after the experiment. The picture to the right is taken with petrographic microscope and shows the surface after the process. Process 2 (gravels used on ochred and greased animal skin) has smoothed the surface leaving some hollows and valleys (in blue). (C) Shows the 3D surface of the experimental gravel before the experiment and (D) shows the surface of the same gravel after the experiment. The picture to the right is taken with petrographic microscope and shows the surface after the process. Process 3 (gravels polished with silts from Landry) is the only one to produce striations. (E) Shows the 3D surface of the experimental gravel before the experiment and (F) shows the surface of the same gravel after the experiment. The picture to the right is taken with a metallographic microscope Leica DM2500 M. All treatments produced a strong downward shift on the Z scale.

As expected, all experimental treatments significantly reduced the roughness of the gravels. The tumbling process, entailing impact of the gravels on each other and their contact with the skin, causes a gradual flattening of the surface resulting in a shiny appearance (Fig 20). Abrasion of gravel against Landry silt also produces a shine. At microscopic scale, the tumbled gravels display no striations. Abrasion of gravel with a metallographic polisher covered with silt produces a characteristic shiny oriented wear pattern which retain large concavities unseen on a number of archaeological gravels covered by striations (Fig 20). Gravels tumbled in a barrel coated with untreated animal skin reached Sq values not substantially different from those recorded on gravels tumbled in a barrel covered with skin treated with ochre and fat. Similar Sq values are also seen on facets modified by abrasion against Landry silt. Wear produced by tumbling with untreated skin and abrading on silt, however, display a more intense gloss, reflected by lower Sdr values. Both tumbling with treated and untreated skin and without ochre and fat produce a smoothness comparable to that recorded on archaeological gravels from Fourneau du Diable, Casserole, Pech de la Boissière and Laugerie Haute and stronger than that measured on Landry lustrous gravels. The roughness values recorded on experimentally tumbled and abraded gravels fall outside the variability of gravels from natural accumulations.

### Ethnographic survey results

Uses were first documented at a low level (e.g. “as luck charm”), and then on completion of the sample, our coding system aggregated initial categories into a higher level (e.g. charm, divination etc. as “ritual”), as described in Table 5. Some cultures had many different uses categorized from keyword mentions; roughly a quarter (19 cultures, of which 13 were hunter-gatherers) had no relevant uses despite the mention of the keyword in a paragraph. A small number of uses crossed multiple categories (e.g. medicine and ritual, counting and social coordination). The final judgement considered the immediate functions to be primary.

**Table 5:** Coding scheme for pebble use with representative examples.

Use	Definition	Example
**Ritual**	Use is given as charms, amulets, religious symbols, curse stones, embodiments of spirits/gods, funeral tokens; or otherwise used as a magic ritual	There is a very strong magic power in these stones, and their form is regarded as variable. For instance, if such a stone is left uncovered it may grow legs and run away; so they are worn hanging from the neck in little sacks. [Aranda]
**Games**	Used as game tokens, or in other childhood activities	Thirty-three stones are arranged in a line which may be straight, but is more often curved or crooked. The player must touch each stone and say “Too” for all 33 stones in order, at a high rate of speed. [Kpelle]
**Tool**	Used in hunting, fishing, craft, headshrinking, or as weights	Dressing a hide is generally woman’s work; sometimes men do this work, using woman’s tools, which include a small sack of pebbles held behind the haft of the leg bone scraper to give added force to each push of the scraper removing fat and detritus from the hide. [Innu]
**Weapon**	Stones explicitly thrown at other people in defence or offence	The sling stones were not worked but were selected from waterworn pebbles (ala) of appropriate size. [Samoan]
**Medicine**	Primary functional use given in a medical remedy	Pour over the head of the sufferer an infusion of bark from the top of the trunk of the Bugare. But before pouring the liquid, it must be frothed. This is done by throwing little stones in and mixing it with a stick. [Azande]
**Music**	Used to make musical instruments or noisemakers (including ritual functions)	A woman’s dance rattle contains many small pebbles and the handle passes entirely through the gourd. [Kuna]
**Counting**	Explicit mention as a counting tool	Pebbles are occasionally employed as a mnemonic aid to counting warp threads when weaving; each pebble represents 10 threads. [Aymara]
**Other**	Various uses as ornament, social coordination, genealogical tool etc	He talks about pedigrees. Our expert … gathers a heap of small stones and says: “This is Ancestor Bangáchon and this is Gáyon, his wife”. Meanwhile he places two small stones, side by side in front of his big toes. “They had four children”, he continues … and four stones are put at a short distance of the two first ones. [A]nd so it goes on; names and more names, stones and more stones … [Ifugao]
**None**	Paragraphs contained the words stone* or pebble* but no relevant uses	

In the full sample, ritual uses (as charms, divination, or other magico-religious functions) were the most commonly described (n = 44), followed by games (n = 25) and music (n = 18). A range of other uses accounted for the remainder. Hunter-gatherer societies differed in the most common use (none, n = 13) but followed the trend for music (n = 11), ritual (n = 10) and games (n = 9) as most common (Table 6). Eating/ingestion as one hypothesized source of shine for pebbles was not noted in the sample; some medicinal uses mentioned pebbles placed in a shaman’s mouth but then removed [66]. Table 1 in the supplementary materials gives full information including the bibliographic sources, paragraph text, and main subsistence, for each case. A bag for carrying is mentioned in four cases; polish is mentioned in one. Separate counts are given for the 38 hunter-gatherer cultures of the full sample. ‘Multiples’ indicates if pebbles were found or inferred to be in groups of four or more, with percentages of Yes:No:Unclear (rounded to nearest integer), and it makes it clear that uses of multiples pebbles together is largely dominant.

**Table 6:** Uses of pebbles in 82 cultures of the standard cross cultural sample.

Use	Full sample	Hunter-gatherers	Multiples (%)		
	(n = 82)	(n = 38)	Yes	No	Unclear
**Ritual**	44	10	39	39	22
**Games**	25	9	84	12	04
**Music**	18	11	100	00	00
**Tool**	12	3	67	25	08
**Weapon**	11	4	64	18	18
**Medicine**	9	5	78	22	00
**Counting**	3	1	100	00	00
**Other**	10	5	40	30	30
**None**	19	13	NA		

## Discussion

Polished pebbles have been found repeatedly at archaeological, including ancient, sites [67, 68]. Few are the cases, however, in which complementary analyses were conducted to eliminate the possibility of a natural origin of the polish or, the case of Landry, provide relevant contextual information. A remarkable exception are the polished pebbles found at the Jiahu site, Henan Province, China [69]. At this site, the skeleton of a male individual with the skull missing, dated to c. 9,000 BP, is associated with two seven-hole bone flutes and a turtle shell with polished pebbles inside. The latter is interpreted as a musical instrument to produce a rhythmic sound.

The geoarchaeological study of the Landry site indicates that the spatial distribution of archaeological remains has not been marginally affected by post-depositional disturbances [18]. Landry is currently the only site for which unequivocal information is available on the stratigraphic and the spatial provenance of the lustrous gravels, their spatial relationship to the other archaeological remains, and their cultural affiliation. The lustrous gravels are clearly associated with the other archaeological remains and dispersed within the archaeological layer, which make their attribution to the Solutrean undisputable. They are highly concentrated in an area interpreted as reflecting domestic activities connected to the use of metamorphic rocks. This area has also yielded few incised dolerite blocks and pebbles [18]. For the archaeological sites excavated earlier, only the cultural and, in few cases, the stratigraphic provenance are known. Theoretically, the best available information on the gravel cultural attribution come from Fourneau du Diable [6] where Peyrony is formal in attributing them to both the Gravettian and the Solutrean. The 136 gravels arranged one next to the other to form a rectangle (Fig 4) were apparently found in Gravettian layers, the others in layers attributed to the Upper Solutrean. While a drawing of this chest piece is available, it is difficult at present to assess the reality of this surprising findings, in absence of additional information. At Laugerie Haute the gravels were also apparently retrieved in Upper Solutrean layers; at Pech de la Boissière and Casserole, they were recovered in Solutrean layers. Our spatial analysis has demonstrated that the distribution of lustrous gravels at Landry is not random nor uniform. They are concentrated in an area corresponding to a portion of the zone with the highest density of the other archaeological remains. On the contrary, the non-lustrous gravels are evenly distributed on the excavated area. Unlike the lustrous gravels, all made of white quartz, they are more variable in size, morphology and color. This finding contradicts the hypothesis that the distribution of the lustrous gravels is the result of a natural process and argues in favor of humans being the main or the exclusive accumulation factor. Apparently Landry visitors selected small, round, white gravels and left or disposed them inside their main activity area. Although the lack of direct comparative samples makes it difficult to establish what criteria guided the choice of the gravels at the other archaeological sites, their comparatively low dimensional variability and roundness suggest these were certainly key selection criteria. It can be therefore reasonably inferred that humans were the major gravel accumulation factor at the other sites as well.

At all the analyzed sites, what makes the gravels peculiar is also their shiny appearance. Since the mode of formation of the sedimentary layers is not different at Landry between the concentrations and the areas outside them, there is no reason for thinking that the site formation processes could have affected the gravels inside and outside the concentrations in a different way and create a gloss only on the former. No evidence of chemical alteration or a similar gloss is observed on the remainder of the archaeological remains found inside the concentrations of artifacts [20, 22]. The hypothesis of gastroliths originating from animal carcasses can also be discarded since the lustrous gravels differ in size, morphology and surface condition from such gravels [48, 49]. In addition, microscopic and textural analysis of quartz gravels from the Landry sieving, experimentally modified with a metallographic polisher, rules out the possibility that the action of silt may produce the distinct wear patterns observed on the gravels found in the Landry archaeological concentrations and at other archaeological sites. This supports the hypothesis that differences in the appearance of gravels found inside and outside the concentrations are due to human agency. Textural analysis has identified three significantly different types of surfaces: rough, typical of gravels collected in natural accumulations and in the Landry sieving; glossy, only found on archaeological gravels from sites excavated in the past; moderately glossy, characteristic of the Landry gravels associated with concentrations of cultural remains. Experimental tumbling of Landry gravels collected outside the archaeological concentrations has produced a shine absent in natural accumulations and similar, in terms of roughness, to that of gravels found at archaeological sites excavated in the past and some lustrous gravels from Landry.

In summary, the roughness analysis of natural, experimental and archaeological gravels supports the hypothesis that after being selected by their size and shape, the archaeological gravels were subjected to a human action that has strongly polished them.

The differences in roughness observed between the lustrous gravels from Landry and other archaeological sites may be due to the fact that 1) only the most lustrous gravels were collected in early excavations; 2) at Landry non- or low-gloss gravels were naturally present in archaeological concentrations, which created a sample that included both lustered and non-lustered gravels; 3) the activity that lustered the gravels at Landry was not as extensive as at other sites; 4) their mineralogical composition is different, the Landry gravels could not reach the degree of lustering observed at other sites, where the gravels are composed of other rocks, while being subjected to the same modifications. Selective collection of gravels from older excavations is possible, but two of the collections examined were from recent excavations in which the sediment was systematically sieved with water. The degree of luster of the gravels from these sites is not substantially different from that observed in the ancient collections, which contradicts the hypothesis of selective gathering. The possibility that the rock of which they are composed may determine the lower luster of the Landry gravels seems partly contradicted by the fact that the experimentally tumbled gravels, composed of quartz, attained a degree of luster similar to that observed on the lustered gravels from other archaeological sites. Although it is difficult to rule out the possibility that some of the discarded factors may be a contributing factor to the differences in luster between Landry and the other sites, the most likely hypothesis appears to be that the luster on the Landry gravels is either the result of the same action to which the gravels from the other sites were subjected, but for a shorter period of time, or the result of a slightly different action.

Microscopic analysis of archaeological, natural and experimental gravels provides essential additional elements for documenting and interpreting the differences between these groups. On the one hand, it confirms that the wear pattern present on archaeological gravels is absent on natural gravels, which further supports its anthropogenic origin. On the other hand, it reveals that the shine observed on the archaeological gravels, similar in terms of roughness values, include in reality at least two major categories of wear, one characterized by the presence of striated areas and the other by their absence, incipient micro fractures and large homogeneously lustered areas with rare crisscrossing striations. The first category results from directional rubbing of the gravels against a surface rich in abrasive particles of variable size, the second from repeated contact with objects of same hardness than the gravels in an environment with very few abrasive elements. This last wear pattern is comparable to that observed on tumbled gravels, which show, however, as documented by texture analysis, a slightly less developed shine. These two wear patterns are consistent with 1) intentional rubbing of the gravels against a variety of slightly abrasive materials with or without the adding or abrasive particles, and 2) prolonged tumbling of numerous gravels in leather containers to polish or transport the gravels. The differences observed in the first wear pattern can be attributed to the materials against which the gravels were rubbed at different archaeological sites, the motions involved and the state of preservation of the wear pattern. The local disappearance of striations on some gravels may result from transport of previously intentionally polished gravels or taphonomic processes. The variability observed in the second wear pattern may have resulted from the type and treatment of the soft materials used for the containers in which the gravels were tumbled or transported.

The long duration of the tumbling experiments necessary to produce a luster similar to the one observed on the archaeological specimens seems to contradict the hypothesis that sporadic contact with soft material or human skin, for example when handling the gravels, may have produced such a developed luster. In summary, results from textural and microscopic analyses concur to identify the lustrous appearance of the archaeological gravels as resulting from intentional polishing, producing oriented striations, probably aimed at making the gravels shinier, and their transport. This hypothesis is consistent with results of texture analysis indicating that although not significantly different from that recorded on the experimentally tumbled gravels, the luster on most archaeological collections reach substantially higher degrees.

These results do not allow us to identify with certainty the function for which the gravels were intended. The luster may, for example, be the result of polishing by abrasion to increase the gravels’ lustrous appearance tumbling in leather containers to obtain a similar effect or transporting the gravels from one site to another. Intentional polishing may have been instrumental, for example, to create visually catching ornaments, as suggested by Peyrony or to use the gravel to play. It is less probable that gravels were intentionally polished with the aim of putting them in a container to produce a rhythmic sound. The actual function may have produced little or no luster.

It is worthwhile for this reason to turn to the ethnographic and archaeological record. The former may inform us on the uses of gravels in different traditional human cultures. The latter may identify cases in which contextual information would allow us to understand the function that gravels had in past populations. Both records open the possibility of analyzing in the future, with the same methodologies applied in our study, ethnographic and archaeological gravels and compare the results of these analyses with ours.

Our ethnographic survey shows that the most common functions for small pebbles are magico-religious ritual uses, and as game pieces. Worldwide, pebbles are used as talismans, amulets, and charms [70–72] (see S1 Table for examples), appear in divination or sorcery kits [73, 74], and often embody spirits and ancestors [75–77]. When used in games, pebbles are most often in multiples and handled frequently, including by children [78–81]. The use of gravels in musical instruments mimics experimental tumbling and can obviously produce the same effects: in our survey we noted drum/rattle containers made from dried fruit [82], cocoons [83], animal shells [84], birch bark [85], as well as hides and gourds [86]. Further uses include tools or weapons, elements in medicinal treatments, ornaments, and in counting (see S1 Table for examples). All these functions involve the transport of gravels, which can produce their gradual polishing. A use for games or shamanism/divination also implies a more or less prolonged handling, which can contribute to their polishing–possibly, in the case of games, from hide/leather surfaces on which pieces are scattered. Some of these functions (games, music, medicine, and counting) are more consistent with the use of many gravels than others (weapons, tools and ‘other’ uses are often single items), but ritual uses can involve single or multiple stones with equal likelihood (Table 6).

While many of the ethnographically attested functions imply a choice of gravels on the basis of size, shape, weight and raw material criteria, exact remarks on size were uncommon in the sources. Anthropologists occasionally noted qualitative features such as color or shape e.g. “marble-like stones” [87], “preferably those of a black color” [88]. However, these remarks were too infrequent (only 12/152 cases) to support generalizations regarding specific functions for the archaeological gravels studied in our work. It is however possible that the smaller and rounder gravels from the Landry and Casserole sites had another function than the larger gravels of the Fourneau du Diable, Laugerie Haute and Pech de la Boissière. However, this is only partially consistent with the result of the microscopic and, in particular, textural analysis, which rather highlights a difference between Landry alone and the other sites. By ethnographic analogy, ritual and gaming uses are most likely, with the uniformity of the gravels and intentional polishing suggesting a deliberate aesthetic preference. Although our cross-cultural survey indicates the use of gravels in counting systems is relatively uncommon (in our sample, 3 of 128 uses or 2.5%), their use for other functions (games, ornaments, divination etc.) certainly implies their approximate and, especially in the case of games, possibly precise quantification. In both cases, the creation of a cultural innovation involving the use of small, visually homologous objects may have played an adjunct role in the complexification of modalities for precise quantification (verbal, finger counting, notational) or may have been a reflection of contemporary or prior advances in these fields [89–91].

## Conclusion

The results of our study indicate that there are means to distinguish objects modified by natural processes from artifacts minimally modified by humans. Use-wear analysis has been widely applied to prehistoric artefacts to identify their function. However, this approach has been implemented on artefacts recognized as the outcome of human agency. In our case, instead, we have faced the challenge of identifying such agency exclusively from surface modification. The chosen means to pursue this endeavor involved evaluation of contextual data, creation of natural and experimental frames of reference, and characterization of natural, experimental and archaeological material using multiple, adapted analytical tools. These analyses leave no doubt: the lustrous gravels discovered in several Gravettian and Solutrean sites in southwestern France were lost or abandoned in these sites after having been selected on the basis of size and shape criteria and having been involved in specific activities that produced their lustrous appearance. These gravels were not discovered in older Gravettian layers during the numerous excavations conducted in the region despite the fact that many of these excavations were conducted in recent times, with modern excavation techniques and by archaeologists aware of the possibility of discovering glossy gravels in Upper Palaeolithic archaeological layers. This leads us to conclude that the activity in which the gravels were involved, which is impossible to identify precisely but could involve a long-time use or curation in a leather bag, reflect a cultural innovation that developed during the Gravettian period and continued into the Solutrean period at the least.

## Supporting information

S1 Table Ethnographic data surveyed from sources in the electronic human relations area files. Region, Culture, Outline of World Cultures (OWC) code, Subsistence Type*, Source, Page, Focus Date, Outline of Cultural Materials (OCM) Subject, and Paragraph are taken direct from eHRAF. Focus Date is the earliest given in the source, or when not given, the publication date. Use denotes a low-level classification of the use. Coded Use is the final code given in the text. Multiples refers to if pebbles were present in multiples of four or more at one time (Yes, No, Unclear). Notes carries any pertinent information about bags or other carrying items, and qualities of the stones (colour, shape etc). Subsistence Type: AP Agro-Pastoralists, HG Hunter-Gatherers, HC Horticulturalists, IA Intensive Agriculturists, OSC Other Subsistence Combinations, PA Pastoralists, PHG Primarily Hunter-Gatherers.
(XLSX)

S2 Table Results of Kruskal-Wallis statistical tests for the different morphometry and roughness parameters we tested. R script and data to produce Figs 7–11, 17 and 19 are available online at the OSF repository https://osf.io/9wpn7/.
(XLSX)
